# Return-to-work for multiple jobholders with a work-related musculoskeletal disorder: A population-based, matched cohort in British Columbia

**DOI:** 10.1371/journal.pone.0193618

**Published:** 2018-04-03

**Authors:** Esther T. Maas, Mieke Koehoorn, Christopher B. McLeod

**Affiliations:** Partnership for Work, Health and Safety, School of Population and Public Health, University of British Columbia, Vancouver, British Columbia, Canada; Monash University, AUSTRALIA

## Abstract

**Introduction:**

Multiple jobholders (MJHs) have a higher risk of injury compared to single jobholders (SJHs), but it is unknown if return-to-work (RTW) after a work injury is affected by multiple jobholding. This study examined the association between multiple versus single jobholding and time to RTW for workers with a work-related musculoskeletal disorder (MSD).

**Methods:**

We used administrative workers’ compensation data to identify injured workers with an accepted MSD lost-time claim between 2010–2014 in British Columbia, Canada (n = 125,639 SJHs and 9,029 MJHs). The outcome was days until RTW during twelve months after the first day of time-loss. The MJH and SJH cohorts were balanced using coarsened exact matching that yielded a final matched cohort of 8,389 MJHs and 8,389 SJHs. The outcome was estimated with Cox regression, using piecewise models, and the hazard ratios were stratified by type of MSD, a serious injury indicator, gender, weekly workdays preceding MSD, and wage categories.

**Results:**

MJHs were less likely to RTW compared to SJHs within the first six months after the first time-loss day, with greater and longer lasting effects for males, workers with a serious injury, and a higher wage. No difference between MJHs and SJHs was found for workers who had a six- or seven-day work week preceding MSD, for workers with dislocations, and for workers who were still off work after six months.

**Conclusions:**

Overall, MJHs with a workweek of maximum five days are disadvantaged compared to SJHs in terms of RTW following a work-related MSD within the first six months after the first time-loss day. This difference might be caused by more precarious job contracts for MJHs that challenges RTW because of lack of support for modified work, higher workload, and reduced likelihood that MJHs file a workers’ compensation claim. Despite adjusting for type of MSD, severity of injury and occupation, the differences persisted for the vast majority of the study sample.

## Introduction

Workers who hold multiple jobs are a growing segment of the workforce in Canada [1,2]. In 2016, 132 100 workers in British Columbia (BC), Canada, were multiple jobholders (MJHs), representing 5.5% of the total BC labour force (N = 2 412 700) [1,3]. Similar numbers are shown for the rest of Canada and in the United States [4].

Multiple jobholding can include having multiple full-time jobs, multiple part-time jobs, self-employment combined with a full- or part-time job, or any other combination thereof. Two main incentives for working in multiple jobs relate to: 1) low-income workers seeking to maximize income through working longer hours and combining part-time jobs, and 2) high-income workers working across different organizations where specialist skills may be in demand [5–8]. Women with multiple jobs are more likely to work at multiple part-time jobs, while men with multiple jobs are more likely to work full-time at a primary job and part-time at a secondary job [8]. Although similar numbers of men and women held multiple jobs in the United States in 2009, the multiple jobholding rate for women (5.6%) was higher than that for men (4.8%) [9]. In some jurisdictions such as the United States, MJHs are more likely to be higher educated, work more than 50 hours per week, and work in a service occupation, compared to single jobholders (SJHs) [8].

Working in more than one job may jeopardize a worker’s health status [10]. A recent American study found that workers with more than one job in a one-week period had a higher risk of injury than SJHs. This risk remained elevated even after they controlled for hours worked [8]. Two other recent studies in the United States have also shown an increased risk of injury for MJHs: an elevated rate of work-related fatalities for MJHs was reported in Kentucky, and an elevated rate and severity of injury was reported for adolescent MJHs in Wisconsin [11,12]. This could be due to long work hours, long daily commutes, multiple shifts, and less sleep and leisure time; all factors that may increase the risk of fatigue and injury for MJHs [8].

In contrast to the higher injury rates, being a MJH was related to a 77% lower risk of work-related sickness absence, and a 54% lower risk of all-cause sickness absence, compared to workers with one job [13]. This finding remained consistent when controlling for higher exposure levels due to increased work time across multiple jobs. The authors suggest that individuals with multiple jobs may be less likely to file a workers’ compensation claim, perhaps due to greater work engagement, precarious work situations and worry about repercussions, or more resilience to adverse health exposures [13–15].

MJHs are generally underrepresented in research. The effect of working multiple jobs on injury has been explored in few studies, mostly in secondary analyses, and the effect of working multiple jobs on the likelihood of return-to-work (RTW) after injury is unknown. The current study focused on comparing RTW among MJHs and SJHs with work-related musculoskeletal disorders (MSDs), because the impact of MSDs on workers, employers, workers’ compensations systems, and society is significant, especially in the aging Canadian workforce. MSDs account for the highest disability costs due to productivity losses in Canada, and the burden of disease from MSDs will increase in the coming decades, given the predicted population growth and aging [16].

The study relied on longitudinal administrative health data from BC to examine the association between multiple versus single jobholding and disability duration for workers with a work-related MSD. We hypothesized that MJHs are less likely to RTW compared to SJHs, due to their increased risk of injury combined with being less likely to file a workers’ compensation claim (or only do so for more severe injuries), more precarious employment contracts, and a higher workload. We stratified the results by type of MSD, a serious injury indicator, gender, weekly workdays preceding MSD, and wage, because results were expected to differ by these factors.

## Materials and methods

### Study design

This matched cohort study was implemented according to the STrenghtening the Reporting of OBservational studies in Epidemiology (STROBE) statement for reporting observational studies [17,18]. More information about the matching is described in the paragraph ‘Matching MJHs and SJHs’. Accepted work-related MSD lost-time claims filed between January 1, 2010 and December 31, 2014 were identified from administrative health data from WorkSafeBC, the provincial workers’ compensation system [19]. This database contains injury information, demographic variables, employer information, pre-injury wages, and occupation classification. The claims database was linked to RTW calendar data, which includes RTW status information at a daily level. The follow-up period was restricted to a maximum of one year, measured as 52 weeks from the first recorded time-loss day. The Behavioral Research Ethics Board at The University of British Columbia approved the study (Certificate no. H15-00779).

### Jurisdictional context

In BC, Canada, workers who experience a recognized work-related injury or disease are provided with disability benefits, medical aid and rehabilitation services by WorkSafeBC, the provincial workers’ compensation system. WorkSafeBC, funded through employer-paid insurance premiums, provides short-term disability wage replacement to injured workers with the goal to return workers to work in a timely manner. Wage-loss benefits compensate workers who lose pay due to a work-related injury or illness. Short-term wage-loss benefits are 90% of the net annual earnings, and are provided for the first ten weeks after injury. Most wage-loss claims do not extend past ten weeks. For those that do, long-term wage-loss compensation is applied. This is generally based on earnings for the past twelve months before injury, after which the wage-loss rate will be calculated. For most injured workers, the wage-loss rate is approximately 90% of their net weekly earnings. Benefits continue until a worker is able to participate in modified work or return to usual duties.

This study focuses on injured workers during the period in which they are provided short-term or long-term wage-loss disability benefits, referred to here as work-related sickness absence during the first twelve months of an injury claim.

### Population

The cohort was restricted to the first MSD work disability claim per worker in the study period. Claimants were excluded from the study for the following reasons:

1. Exclusions based on cohort definitions:
  - Claim is not related to a classifiable MSD (defined using International Classification of Diseases, Ninth Revision, Clinical Modification (ICD-9-CM) codes and National Work Injury Statistics Program (NWISP) WorkSafeBC Nature of Injury codes)
  - Age <15 or ≥65 years
  - Claim is incomplete (< one year follow-up information is available)
  - Claim is related to previous non-MSD claims
  - Claim from self-insured industry sectors
  - Claim for a work-related fatal injury
2. Exclusions based on missing data on firm size, industry, wage, or gender information
3. Exclusions based on claims with less than one day off work

A total of 150 539 unique MSD claims were identified between January 1, 2010 and December 31, 2014, including 140 371 SJHs and 10 168 MJHs.

#### Selection of multiple jobholders (MJHs)

MJHs were defined as workers with a work-related MSD time-loss claim for which the claim was associated with multiple employers. The eligibility restrictions were conducted in three stages: (1) excluding claims based on cohort definitions (N = 1 031); (2) excluding claims based on missing data (N = 43); and (3) restricting the cohort to workers with at least one day off work (excluding N = 65). This led to a final cohort of 9 029 MJHs.

#### Selection of single jobholders (SJHs)

SJHs, for whom the RTW trajectory involved only one employer were selected as follow: (1) excluding claims based on cohort definitions (N = 13 422); (2) excluding claims based on missing data (N = 687); and (3) restricting the cohort to workers with at least one day off work to ensure a RTW trajectory (excluding N = 623). This led to a final cohort of 125 639 SJHs.

**Fig 1:**
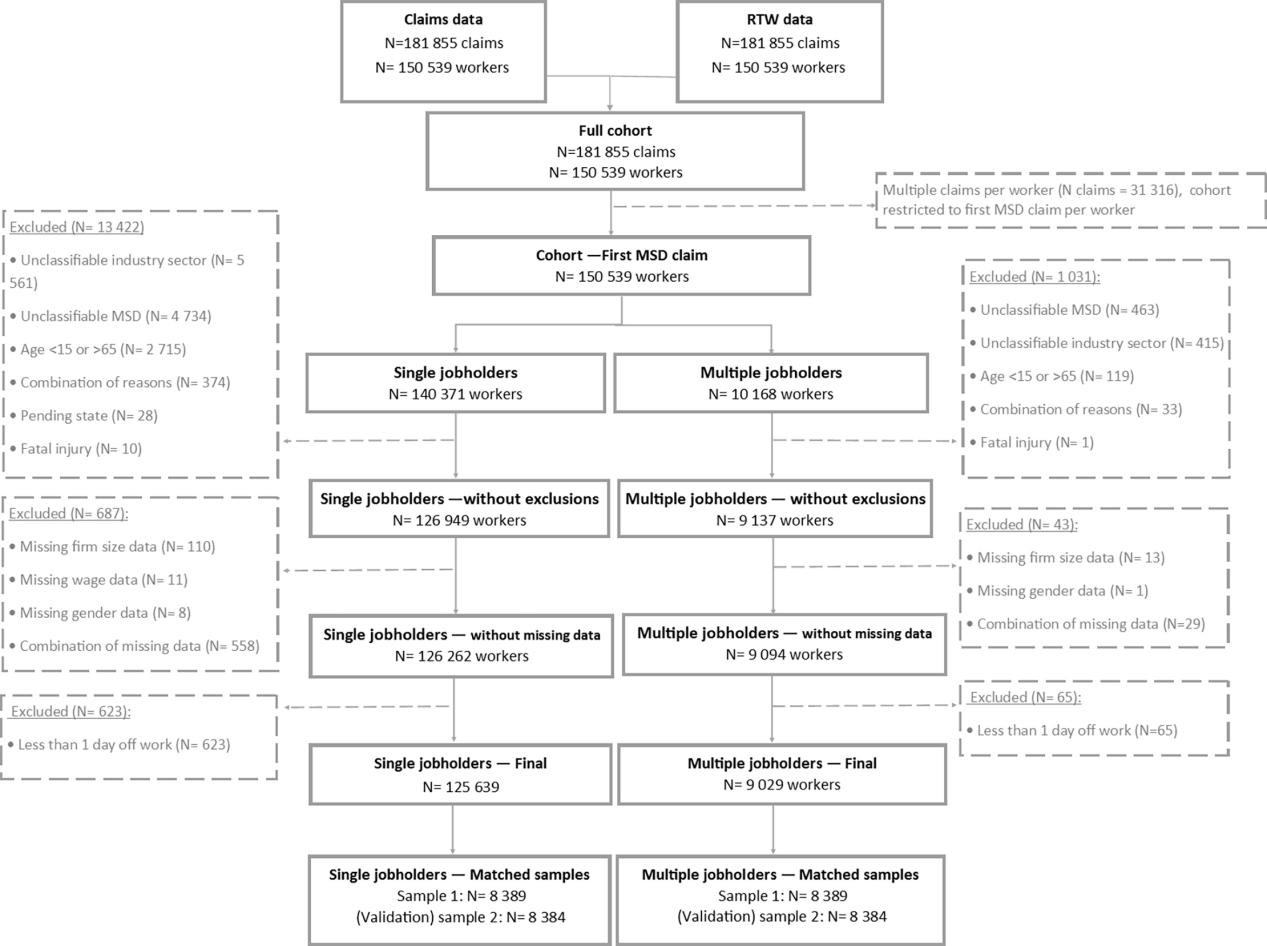
Flow chart, 2010–2014 study cohort.

Detailed information on the exclusion criteria is shown in Fig 1.

### Outcomes

The outcome variable was time until RTW during twelve months following the first time-loss day, and was defined as the period (in calendar days) between the first time-loss day and RTW as the end event of the claim.

### Musculoskeletal disorders (MSDs)

MSDs were categorized into nine major categories using the Barell matrix [20] (for musculoskeletal injuries: sprains/strains, fractures, dislocations) and ICD-9-CM codes (for musculoskeletal diseases: dorsopathies and rheumatism (excluding the back)). Sprains/strains and fractures were divided into three body regions; (i) head&neck/spine/back/torso; (ii) upper extremities; (iii) lower extremities. Disorders not mapping to one of these nine categories were excluded from the cohort because their numbers were too small to be an independent category.

### Covariates

Analyses were adjusted for the following covariates measured at the start of time-loss for an MSD:

- Gender
- Age (15–24, 25–34, 35–44, 45–54, 55–64 years)
- Annual wage (<$20 000, $20 000-$39 999, $40 000–59 999, >$59 999 (Canadian dollars))
- Occupation, classified by ten categories according to Statistics Canada’s Standard Occupational Classification [21]
- Industry sector, classified by seven categories according to the WorkSafeBC industry classification structure [22]
- Size of the workers’ firm measured as fulltime-equivalent (FTE) workers employed by the firm (<20, 20–99, 100–499, 500–999, >999 FTE)
- History of prior claims (yes/no): at least one accepted claim in the preceding ten years to the MSD claim
- Weekly workdays preceding MSD eligible for wage replacement (0–7), categorized in five or less weekly workdays (or typical workweek) and six or seven weekly workdays (more than typical workweek)
- Serious injury indicator (Y/N)

### Matching MJH and SJH

When estimating causal effects using observational data, it is desirable to replicate a randomized experiment as closely as possible by obtaining exposed and non-exposed groups with similar covariate distributions. This goal can be achieved by choosing well-matched samples of the original groups, and thereby reducing bias due to the covariates [23]. SJHs and MJHs were matched to estimate the impact of having more than one job on RTW for workers with a work-related MSD. Baseline characteristics were compared with the Student’s T-test, the Mann-Whitney U-Test, or the Chi^2^ Test, as appropriate. A p-value of <0.05 indicated that a baseline characteristic was significantly different between MJHs and SJHs. Using a coarsened exact matching (CEM) strategy, MJHs and SJHs were matched for analyses on differing baseline characteristics [24,25]: type of MSD, gender, age, annual wage, occupational classification, and industry sector. Firm size and prior claims were not used for matching, because there was no difference in the distribution between the MJHs and SJHs for these variables. Weekly workdays preceding MSD and the serious injury indicator were not used for matching, because of a possible mediating effect of workdays and injury severity in the relationship between multiple jobholding and RTW. The rationale for using CEM as a matching strategy is to identify similar groupings of variables so that the covariate values are the same between MJHs and SJHs in each matching stratum [26]. All MJHs and SJHs were sorted into strata, each of which had identical values for all matched covariates within their stratum. Within a given stratum, a SJH observation was matched using random assignment to a corresponding MJHs observation, based on the MJHs characteristics. Excess and non-matched SJH observations were discarded as were MJH observations that did not have a corresponding match within a stratum. The aim of matching was to find balance across the multidimensional distribution of covariates of the groups. This reduces the degree of dependence on the estimation model of the outcome variable and reduces estimation bias [27].

One-to-one matching was applied and all MJHs were matched twice to half of the SJHs. The second matched cohort was used to test the reproducibility of the results from the first matched sample and validate the findings. Firstly, all MJHs (N = 9 029) were matched with SJHs drawn from 50% of the full SJH sample (N = 62 819). Secondly, all MJHs (N = 9 029) were matched with SJHs drawn from the other 50% of the full SJH sample (N = 62 820). This matching strategy resulted in 8 389 MJHs and 8 389 SJHs in the first matched sample, and 8 384 MJHs and 8 384 SJHs in the second matched sample (validation cohort), representing 92.9% and 92.8% of all eligible MJHs.

Covariate balance between the matched SJH and MJH sample was assessed using a multivariable imbalance measure (L1). L1 ranges from zero to one and tends toward zero the more the two distributions (in this case SJHs and MJHs) overlap. It is a relative measure and its meaning depends on the dataset and the selected covariates [26].

### Statistical analysis

Descriptive statistics (frequency counts and proportions) were applied to describe the total study cohort, and to compare baseline characteristics between the unmatched and matched cohorts.

Cumulative incidence proportions (CIP) showed the percentages of individuals who returned to work within one year after injury CIP is calculated over full data and evaluated at six timeframes over the year: 1–30 days, 31–60 days, 61–90 days, 91–180 days, 181–270 days, and 271–365 days.

Cox regression, using piecewise models, was used to examine the difference in disability duration measured in days until RTW between SJHs and MJHs by calculating hazard ratios (HR) with 95% confidence intervals (CI) over the year after the first time-loss day. To handle non-proportionality and show multiple effects over time of multiple jobholding on the probability to RTW, piecewise models with six outcomes were estimated: RTW 30, 60, 90, 180, 270, and 365 days after the first time-loss day. These timeframes are used as defined by the Association of Workers’ Compensation Board of Canada (AWCBC). The HRs were stepwise adjusted for all study covariates. Additionally, the HRs were stratified by type of MSD, a serious injury indicator, gender, weekly workdays preceding MSD, and wage for possible effect modifying effects. Statistical analyses were performed using Stata 14 (Stata Corp LP, College Station, TX, USA).

## Results

### Sample characteristics

The unmatched cohort comprised 125 639 (93.29%) SJHs and 9 029 (6.71%) MJHs. Back sprains and strains were the most common disorder type (43.28% of the SJHs, and 34.08% of the MJHs), while dislocations (1.65% of the SJHS, 2.69% of the MJHs) were the least common. The SJHs cohort comprised more men (62.30%), while gender was balanced in the MJHs cohort (51.01% men). The mean age was 41.0 years (sd 12.30) in both groups. SJHs had a median annual wage of $41 459 (Canadian dollars) (Interquartile range (IQR): $29 614-$58 134), compared to $45 995 (IQR: $32 165-$62 676) for the MJHs (income from all jobs). Most SJHs (41.77%) and MJHs (55.58%) worked in the services sector, but the largest occupation category for SJHs was trades (38.03%), while for MJHs this was sales and services (27.88%). On average, 25% of both SJHs and MJHs had a history of prior claims. SJHs worked in a firm with a median of 136 FTE workers (IQR: 26–1157 FTE), and MJHs 153 FTE workers (IQR: 27–1184 FTE).

After improving covariate balance by matching, the matched cohorts resulted in equal distributions between the SJHs and MJHs on all covariates (see Table 1). The multivariable imbalance measure L1 improved from 0.398 to 0.000 by CEM, indicating that balance in the matched cohorts could not be improved further.

**Table 1:** Matched variables of single- and multiple jobholders with an accepted MSD lost-time claim between 2010–2014.

	UNMATCHED SAMPLE		MATCHED SAMPLE	
	Single jobholders	Multiple jobholders	Single jobholders	Multiple jobholders
	N= 125 639 (93.29%)	N= 9 029 (6.71%)	N= 8 389 (50.00%)	N= 8 389 (50.00%)
**Musculoskeletal disorder**	** **			
- Upper Extremity Sprains & Strains	23 160 (18.43)	1 865 (20.66)	1 777 (21.18)	1 777 (21.18)
- Lower Extremity Sprains & Strains	21 120 (16.81)	1 610 (17.83)	1 523(18.15)	1 523(18.15)
- Back^b^ Sprains & Strains	54 379 (43.28)	3 077 (34.08)	3 025 (36.06)	3 025 (36.06)
- Upper Extremity Fractures	6 056 (4.82)	654 (7.24)	539 (6.43)	539 (6.43)
- Lower Extremity Fractures	4 200 (3.34)	562 (6.22)	460 (5.48)	460 (5.48)
- Torso^c^ Fractures	2 477 (1.97)	216 (2.39)	166 (1.98)	166 (1.98)
- Dislocation	2 076 (1.65)	243 (2.69)	173 (2.06)	173 (2.06)
- Dorsopathies	6 300 (5.01)	388 (4.30)	342 (4.08)	342 (4.08)
- Rheumatism (excluding the back)	5 871 (4.67)	414 (4.59)	384 (4.58)	384 (4.58)
**Gender**				
- Male	78 267 (62.30)	4 612 (51.08)	4 279 (51.01)	4 279 (51.01)
- Female	47 372 (37.70)	4 417 (48.92)	4 110 (48.99)	4 110 (48.99)
**Age**				
- 15–24	14 580 (11.60)	900 (9.97)	803 (9.57)	803 (9.57)
- 25–34	26 683 (21.24)	1 901 (21.23)	1 763 (21.02)	1 763 (21.02)
- 35–44	28 438 (22.63)	2 148 (23.79)	2 011 (23.97)	2 011 (23.97)
- 45–54	35 080 (27.92)	2 767 (30.65)	2 608 (31.09)	2 608 (31.09)
- 55–64	20 858 (16.60)	1 313 (14.54)	1 204 (14.35)	1 204 (14.35)
**Annual wage ($ Canadian)**				
- <$20.000	12 431 (9.89)	773 (8.56)	657 (7.83)	657 (7.83)
- $20.000-$39.999	46 813 (37.26)	2 789 (30.89)	2 646 (31.54)	2 646 (31.54)
- $40.000-$59.999	37 626 (29.95)	2 963 (32.82)	2 788 (33.23)	2 788 (33.23)
- >$59.999	28 769 (22.90)	2 504 (27.73)	2 298 (27.39)	2 298 (27.39)
**Occupation**^**a**^				
- Management	3 334 (2.65)	169 (1.87)	127 (1.51)	127 (1.51)
- Business^d^	5 596 (4.45)	356 (3.94)	289 (3.44)	289 (3.44)
- Natural sciences^e^	2 381 (1.90)	117 (1.30)	84 (1.00)	84 (1.00)
- Health	15 294 (12.17)	1 659 (18.37)	1 627 (19.39)	1 627 (19.39)
- Social science^f^	5 748 (4.58)	677 (7.50)	617 (7.35)	617 (7.35)
- Recreation^g^	1 876 (1.58)	347 (3.84)	250 (2.98)	250 (2.98)
- Sales, service	28 549 (22.72)	2 517 (27.88)	2 365 (28.19)	2 365 (28.19)
- Trades^h^	47 778 (38.03)	2 393 (26.50)	2 331 (27.79)	2 331 (27.79)
- Primary industry	5 431 (4.32)	305 (3.38)	256 (3.05)	256 (3.05)
- Manufacturing^i^	9 652 (7.68)	489 (5.42)	443 (5.28)	443 (5.28)
**Industry**^**a**^				
- Primary resources	4 825 (3.84)	296 (3 28)	240 (2.86)	240 (2.86)
- Manufacturing	15 510 (12.34)	756 (8.37)	704 (8.22)	704 (8.22)
- Construction	17 641 (14.04)	876 (9.70)	822 (9.80)	822 (9.80)
- Transportation^j^	11 946 (9.51)	691 (7.65)	636 (7.58)	636 (7.58)
- Trade	17 697 (14.09)	921 (10.20)	856 (10.20)	856 (10.20)
- Public sector	5 538 (4.41)	471 (5.22)	353 (4.21)	353 (4.21)
- Service sector	52 482 (41.77)	5 018 (55.58)	4 778 (56.96)	4 778 (56.96)
**MULTIVARIATE L1 DISTANCE**^**K**^		0.398		0.000

^a^ Most responsible firm for the claim of multiple jobholders

^b^ Back, head, neck, spine and torso

^c^ Torso, back, neck, spine and head

^d^ Business, finance and administration

^e^ Natural and applied sciences, related occupations

^f^ Social science, education, government, service and religion

^g^ Recreation, arts, culture and sport

^h^ Trades, transport, equipment operators and related occupations

^I^ Manufacturing, processing and utilities

^j^ Transportation and warehousing

^k^ L1 has a relative magnitude that ranges from 0 to 1 and tends to 0 the more the SJHs and MJHs overlap

Table 1 shows the descriptive statistics of the unmatched and first matched cohort of SJHs and MJHs in detail for comparison purposes of the original population and the matched analytic sample. The descriptive statistics of the second matched validation cohort are presented in S1 Table.

### Likelihood to RTW for MJHs and SJHs

A total of 79.39% of MJHs RTW within one year after the first time-loss day, compared to 86.26% of the SJHs. SJHs in the matched cohort only represent the SJHs matched on MJHs characteristics, and do not represent SJHs in general.

The univariate Cox regression model to predict RTW confirmed a reduced likelihood to RTW for MJHs until 180 days after the first time-loss day (crude Hazard Ratio (HR): 0.81; 95% Confidence Interval (CI) 0.75–0.88), with the largest effect within the first 30 days (crude HR: 0.63; 95%CI 0.59–0.66) (Table 2). This effect remained consistent controlling for demographic and work-related factors (adjusted model 1 HR 91–180 days: 0.82; 95%CI 0.75–0.86); and controlling additionally for weekly workdays preceding MSD (adjusted model 2 HR 91–180 days: 0.85; 95%CI 0.78–0.92). From 181–270 days after the first time-loss day, MJHs had a small increased likelihood to RTW compared to SJHs (adjusted model 2 HR 181–270 days: 1.31; 95%CI 1.14–1.49), and no difference between MJHs and SJHs was found between 271–365 days (HR 271–365 days: 1.04; 95%CI 0.86–1.26). This represents only 10% of the MJHs and 15% of the SJHs who RTW after 180 days.

**Table 2:** Likelihood to return to work for multiple jobholders and single jobholders on sickness absence due to an MSD during 1 year follow-up.

Days after the first time-loss day	*Workers not returned to work at end of time frame(Multiple (N = 8 389) vs. single jobholders (N = 8 389))*	*CIP %*	*Crude model(HR (95% CI))*	Adjusted model1* *(HR (95% CI))*	Adjusted model 2** (*HR (95% CI))*
0–30	Multiple (N = 5 697) vs. single jobholders (N = 4 538)	32.56 vs. 46.30	0.63 (0.59–0.66)	0.63 (0.60–0.66)	0.66 (0.63–0.70)
31–60	Multiple (N = 4 613) vs. single jobholders (N = 3 421)	45.23 vs. 59.57	0.73 (0.68–0.79)	0.74 (0.69–0.80)	0.77 (0.70–0.83)
61–90	Multiple (N = 3 798) vs. single jobholders (N = 2 651)	55.01 vs. 68.61	0.77 (0.70–0.85)	0.78 (0.70–0.86)	0.81 (0.73–0.89)
91–180	Multiple (N = 2 597) vs. single jobholders (N = 1 668)	69.17 vs. 80.21	0.81 (0.75–0.88)	0.82 (0.75–0.89)	0.85 (0.78–0.92)
181–270	Multiple (N = 1 998) vs. single jobholders (N = 1 341)	76.24 vs. 84.05	1.22 (1.06–1.39)	1.25 (1.09–1.44)	1.31 (1.14–1.49)
271–365	Multiple (N = 1 731) vs. single jobholders (N = 1 153)	79.39 vs. 86.26	0.95 (0.78–1.14)	0.98 (0.81–1.19)	1.04 (0.86–1.26)

CIP: cumulative incidence proportion, shows the percentages of individuals having returned to work within one year after injury. CIP is calculated over full data and evaluated at indicated times; it is not calculated from aggregates shown at left. HR: Hazard ratio; CI: Confidence interval

* Adjusted for MSD, gender, age, occupation, industry, previous claims, and firm size

** Adjusted for variables in model 1, and weekly workdays preceding MSD eligible for compensation benefits

### Likelihood to RTW for MJHs and SJHs, stratified by MSD

Table 3 provides proportions of MJHs and SJHs who RTW for each MSD separately. The likelihood to RTW was lower for the MJHs compared to the SJHs for the first 60 days after the first time-loss day for all MSDs except dislocations (no difference) and dorsopathies (MJHs were less likely to RTW compared to SJH only until 30 days). For workers with back sprains and strains, the likelihood to RTW was lower for MJHs compared to SJHs until the first 90 days after the first time-loss days; and until 180 days for workers with upper extremity sprains and strains, and fractures. No difference between MJHs and SJHs was found in the second 180 days after the first time-loss day, or MJHs were slightly more likely to RTW than SJHs. In sum, as the majority of workers RTW within the first 180 days, most MJHs were less likely than SJHs to RTW across all injury comparisons.

**Table 3:** Likelihood to return to work for multiple jobholders and single jobholders on sickness absence due to an MSD during 1 year follow-up, stratified by MSD.

Days after the first time-loss day	Workers not returned to work at end of time frame	CIP %	Crude model(HR (95% CI))	Adjusted model 1* (HR (95% CI))	Adjusted model 2** (HR (95% CI))
**Upper extremity sprains & strains** (Multiple (N = 1 777) vs. single jobholders (N = 1 777))					
0–30	Multiple (N = 1 275) vs. single jobholders (N = 966)	28.70 vs. 45.98	0.54 (0.48–0.60)	0.54 (0.48–0.61)	0.58 (0.52–0.65)
31–60	Multiple (N = 1 051) vs. single jobholders (N = 751)	41.02 vs. 58.08	0.74 (0.62–0.89)	0.75 (0.62–0.91)	0.79 (0.66–0.96)
61–90	Multiple (N = 888) vs. single jobholders (N = 609)	50.37 vs. 65.79	0.84 (0.67–1.05)	0.85 (0.68–1.06)	0.90 (0.72–1.12)
91–180	Multiple (N = 627) vs. single jobholders (N = 386)	64.77 vs. 78.33	0.75 (0.63–0.90)	0.77 (0.64–0.92)	0.80 (0.67–0.96)
181–270	Multiple (N = 505) vs. single jobholders (N = 313)	71.64 vs. 82.44	1.04 (0.78–1.39)	1.07 (0.81–1.44)	1.13 (0.85–1.52)
271–365	Multiple (N = 440) vs. single jobholders (N = 269)	75.24 vs. 84.86	0.92 (0.62–1.35)	0.96 (0.65–1.42)	1.04 (0.70–1.52)
**Lower extremity sprains & strains** (Multiple (N = 1 523) vs. single jobholders (N = 1 523))					
0–30	Multiple (N = 925) vs. single jobholders (N = 697)	39.72 vs. 54.50	0.64 (0.57–0.71)	0.64 (0.57–0.71)	0.66 (0.60–0.73)
31–60	Multiple (N = 748) vs. single jobholders (N = 522)	51.08 vs. 69.16	0.73 (0.58–0.89)	0.73 (0.59–0.91)	0.73 (0.59–0.90)
61–90	Multiple (N = 623) vs. single jobholders (N = 432)	59.29 vs. 79.17	0.97 (0.74–1.27)	0.98 (0.74–1.29)	0.99 (0.75–1.30)
91–180	Multiple (N = 453) vs. single jobholders (N = 298)	70.45 vs. 87.64	0.87 (0.69–1.08)	0.87 (0.69–1.10)	0.90 (0.71–1.13)
181–270	Multiple (N = 325) vs. single jobholders (N = 229)	78.73 vs. 89.22	1.24 (0.93–1.66)	1.25 (0.93–1.67)	1.30 (0.97–1.74)
271–365	Multiple (N = 270) vs. single jobholders (N = 178)	82.34 vs. 89.99	0.76 (0.51–1.12)	0.77 (0.52–1.13)	0.80 (0.55–1.18)
**Back sprains & strains** (Multiple (N = 3 025) vs. single jobholders (N = 3 025))					
0–30	Multiple (N = 1 770) vs. single jobholders (N = 1 404)	43.06 vs. 54.15	0.69 (0.64–0.74)	0.70 (0.65–0.75)	0.74 (0.68–0.79)
31–60	Multiple (N = 1 265) vs. single jobholders (N = 942)	58.47 vs. 69.16	0.84 (0.74–0.95)	0.85 (0.75–0.97)	0.88 (0.78–1.00)
61–90	Multiple (N = 957) vs. single jobholders (N = 640)	68.68 vs. 79.17	0.72 (0.61–0.84)	0.73 (0.63–0.86)	0.76 (0.65–0.89)
91–180	Multiple (N = 586) vs. single jobholders (N = 376)	80.65 vs. 87.64	0.90 (0.77–1.06)	0.94 (0.80–1.09)	0.95 (0.81–1.12)
181–270	Multiple (N = 477) vs. single jobholders (N = 327)	84.26 vs. 89.22	1.51 (1.08–2.13)	1.58 (1.12–2.23)	1.65 (1.17–2.31)
271–365	Multiple (N = 442) vs. single jobholders (N = 305)	85.38 vs. 89.92	1.10 (0.64–1.90)	1.16 (0.67–1.99)	1.21 (0.70–2.09)
**Upper extremity fractures** (Multiple (N = 539) vs. single jobholders (N = 539))					
0–30	Multiple (N = 466) vs. single jobholders (N = 407)	13.91 vs. 25.05	0.52 (0.39–0.69)	0.52 (0.39–0.69)	0.56 (0.42–0.74)
31–60	Multiple (N = 415) vs. single jobholders (N = 315)	23.19 vs. 42.49	0.43 (0.30–0.61)	0.43 (0.30–0.61)	0.45 (0.32–0.64)
61–90	Multiple (N = 341) vs. single jobholders (N = 239)	37.11 vs. 56.59	0.72 (0.52–0.99)	0.72 (0.52–0.99)	0.77 (0.56–1.06)
91–180	Multiple (N = 201) vs. single jobholders (N = 130)	62.89 vs. 76.44	0.84 (0.66–1.09)	0.82 (0.63–1.06)	0.86 (0.66–1.11)
181–270	Multiple (N = 151) vs. single jobholders (N = 100)	72.17 vs. 81.63	1.16 (0.73–1.85)	1.12 (0.71–1.79)	1.12 (0.70–1.79)
271–365	Multiple (N = 122) vs. single jobholders (N = 88)	77.37 vs. 83.67	1.78 (0.89–3.58)	1.75 (0.87–3.52)	1.73 (0.86–3.47)
**Lower extremity fractures** (Multiple (N = 460) vs. single jobholders (N = 460))					
0–30	Multiple (N = 432) vs. single jobholders (N = 383)	6.74 vs. 16.96	0.38 (0.25–0.57)	0.38 (0.25–0.57)	0.42 (0.27–0.64)
31–60	Multiple (N = 408) vs. single jobholders (N = 332)	11.52 vs. 28.48	0.35 (0.21–0.58)	0.36 (0.22–0.59)	0.39 (0.24–0.65)
61–90	Multiple (N = 355) vs. single jobholders (N = 274)	23.04 vs. 40.87	0.72 (0.50–1.05)	0.71 (0.49–1.04)	0.78 (0.53–1.13)
91–180	Multiple (N = 265) vs. single jobholders (N = 166)	43.04 vs. 64.35	0.59 (0.45–0.78)	0.58 (0.44–0.77)	0.62 (0.47–0.82)
181–270	Multiple (N = 197) vs. single jobholders (N = 126)	57.39 vs. 72.83	1.10 (0.73–1.63)	1.12 (0.75–1.66)	1.20 (0.81–1.80)
271–365	Multiple (N = 173) vs. single jobholders (N = 108)	62.39 vs. 76.52	0.84 (0.45–1.59)	0.87 (0.47–1.64)	0.98 (0.52–1.85)
**Torso fractures** (Multiple (N = 166) vs. single jobholders (N = 166))					
0–30	Multiple (N = 139) vs. single jobholders (N = 127)	16.87 vs. 24.10	0.68 (0.42–1.11)	0.67 (0.41–1.09)	0.79 (0.48–1.30)
31–60	Multiple (N = 120) vs. single jobholders (N = 102)	28.31 vs. 39.16	0.66 (0.37–1.21)	0.64 (0.35–1.17)	0.74 (0.40–1.35)
61–90	Multiple (N = 112) vs. single jobholders (N = 84)	33.13 vs. 51.20	0.31 (0.14–0.71)	0.30 (0.13–0.68)	0.33 (0.15–0.76)
91–180	Multiple (N = 96) vs. single jobholders (N = 59)	42.77 vs. 65.06	0.46 (0.24–0.86)	0.41 (0.22–0.80)	0.44 (0.23–0.85)
181–270	Multiple (N = 75) vs. single jobholders (N = 47)	55.42 vs. 72.89	1.01 (0.50–2.01)	1.94 (0.47–1.91)	1.04 (0.52–2.11)
271–365	Multiple (N = 65) vs. single jobholders (N = 40)	60.84 vs. 75.90	1.07 (0.36–3.19)	1.02 (0.34–3.07)	1.16 (0.39–3.51)
**Dislocations** (Multiple (N = 173) vs. single jobholders (N = 173))					
0–30	Multiple (N = 153) vs. single jobholders (N = 139)	13.29 vs. 20.81	0.61 (0.36–1.02)	0.60 (0.35–1.02)	0.58 (0.34–1.00)
31–60	Multiple (N = 132) vs. single jobholders (N = 125)	24.28 vs. 28.32	1.34 (0.66–2.73)	1.33 (0.65–2.71)	1.40 (0.69–2.86)
61–90	Multiple (N = 121) vs. single jobholders (N = 111)	30.64 vs. 36.42	0.72 (0.33–1.58)	0.71 (0.32–1.57)	0.68 (0.30–1.53)
91–180	Multiple (N = 94) vs. single jobholders (N = 78)	46.24 vs. 55.49	0.71 (0.42–1.18)	0.69 (0.41–1.16)	0.75 (0.45–1.26)
181–270	Multiple (N = 73) vs. single jobholders (N = 60)	58.38 vs. 65.90	0.92 (0.49–1.74)	0.91 (0.48–1.72)	1.00 (0.53–1.92)
271–365	Multiple (N = 54) vs. single jobholders (N = 41)	69.36 vs. 76.30	0.81 (0.43–1.55)	0.78 (0.41–1.51)	0.86 (0.44–1.68)
**Dorsopathies** (Multiple (N = 342) vs. single jobholders (N = 342))					
0–30	Multiple (N = 240) vs. single jobholders (N = 168)	30.41 vs. 51.17	0.51 (0.40–0.65)	0.52 (0.41–0.67)	0.55 (0.43–0.71)
31–60	Multiple (N = 202) vs. single jobholders (N = 136)	41.23 vs. 60.53	0.80 (0.50–1.28)	0.81 (0.50–1.31)	0.82 (0.51–1.32)
61–90	Multiple (N = 174) vs. single jobholders (N = 113)	50.00 vs. 67.84	0.80 (0.47–1.35)	0.80 (0.50–1.36)	0.80 (0.47–1.36)
91–180	Multiple (N = 131) vs. single jobholders (N = 73)	62.28 vs. 78.95	0.64 (0.42–1.00)	0.63 (0.41–0.98)	0.66 (0.42–1.02)
181–270	Multiple (N = 100) vs. single jobholders (N = 65)	71.05 vs. 81.29	2.28 (1.05–5.00)	2.23 (1.02–4.89)	2.39 (1.09–5.24)
271–365	Multiple (N = 85) vs. single jobholders (N = 56)	75.15 vs. 83.63	1.13 (0.47–2.70)	1.13 (0.47–2.71)	1.18 (0.49–2.83)
**Rheumatism (excluding the back)** (Multiple (N = 384) vs. single jobholders (N = 384))					
0–30	Multiple (N = 304) vs. single jobholders (N = 251)	21.61 vs. 35.16	0.56 (0.43–0.74)	0.57 (0.43–0.74)	0.60 (0.45–0.78)
31–60	Multiple (N = 274) vs. single jobholders (N = 197)	28.91 vs. 48.96	0.41 (0.26–0.65)	0.41 (0.26–0.66)	0.43 (0.27–0.68)
61–90	Multiple (N = 231) vs. single jobholders (N = 160)	40.10 vs. 58.59	0.82 (0.53–1.28)	0.83 (0.53–1.29)	0.82 (0.52–1.28)
91–180	Multiple (N = 147) vs. single jobholders (N = 107)	61.98 vs. 72.40	1.09 (0.77–1.53)	1.10 (0.78–1.54)	1.11 (0.78–1.57)
181–270	Multiple (N = 100) vs. single jobholders (N = 82)	74.48 vs. 78.91	1.44 (0.89–2.33)	1.43 (0.88–2.32)	1.50 (0.92–2.43)
271–365	Multiple (N = 80) vs. single jobholders (N = 68)	79.17 vs. 82.29	1.19 (0.58–2.43)	1.13 (0.55–2.31)	1.20 (0.59–2.46)

CIP: cumulative incidence proportion, shows the percentages of individuals having returned to work within one year after injury CIP is calculated over full data and evaluated at indicated times; it is not calculated from aggregates shown at left. HR: Hazard ratio; CI: Confidence interval

* Adjusted for MSD, gender, age, occupation, industry, previous claims, and firm size

** Adjusted for variables in model 1, and weekly workdays preceding MSD eligible for compensation benefits

### Likelihood to RTW for MJHs and SJHs, stratified by serious injury

Overall, workers with a serious injury were less likely to return to work compared to workers without a serious injury: 70.44% versus 84.90%. For workers both with and without a serious injury, MJHs were less likely to RTW within the first 180 days after the first time-loss day compared to SJHs (Table 4).

**Table 4:** Likelihood to return to work for multiple jobholders and single jobholders on sickness absence due to an MSD during 1 year follow-up, stratified by serious injury indicator.

Days after the first time-loss day	Workers not returned to work at end of time frame	CIP %	Crude model(HR (95% CI))	Adjusted model 1* (HR (95% CI))	Adjusted model 2** (HR (95% CI))
**No serious injury** (Multiple (N = 7 082) vs. single jobholders (N = 7 307))					
0–30	Multiple (N = 4 483) vs. single jobholders (N = 3 579)	37.25 vs. 51.44	0.64 (0.61–0.67)	0.65 (0.62–0.68)	0.68 (0.65–0.71)
31–60	Multiple (N = 3 484) vs. single jobholders (N = 2 564)	51.04 vs. 65.24	0.74 (0.68–0.81)	0.76 (0.70–0.83)	0.78 (0.71–0.85)
61–90	Multiple (N = 2 814) vs. single jobholders (N = 1 940)	60.54 vs. 73.60	0.78 (0.70–0.87)	0.80 (0.71–0.89)	0.81 (0.73–0.90)
91–180	Multiple (N = 1 893) vs. single jobholders (N = 1 245)	73.37 vs. 83.00	0.88 (0.80–0.98)	0.90 (0.82–1.00)	0.92 (0.84–1.00)
181–270	Multiple (N = 1 472) vs. single jobholders (N = 1 022)	79.25 vs. 86.05	1.27 (1.08–1.50)	1.32 (1.12–1.55)	1.37 (1.16–1.61)
271–365	Multiple (N = 1 287) vs. single jobholders (N = 881)	81.85 vs. 87.94	0.92 (0.73–1.14)	0.96 (0.77–1.19)	1.00 (0.80–1.25)
**Serious injury** (Multiple (N = 1 307) vs. single jobholders (N = 1 082))					
0–30	Multiple (N = 1 215) vs. single jobholders (N = 959)	7.12 vs. 11.55	0.60 (0.46–0.79)	0.61 (0.47–0.80)	0.66 (0.50–0.86)
31–60	Multiple (N = 1 129) vs. single jobholders (N = 857)	13.77 vs. 21.26	0.64 (0.48–0.85)	0.64 (0.48–0.85)	0.69 (0.52–0.92)
61–90	Multiple (N = 984) vs. single jobholders (N = 7 111)	25.02 vs. 34.94	0.73 (0.58–0.92)	0.74 (0.59–0.93)	0.80 (0.63–1.00)
91–180	Multiple (N = 704) vs. single jobholders (N = 423)	46.44 vs. 61.37	0.64 (0.54–0.75)	0.63 (0.54–0.75)	0.67 (0.57–0.79)
181–270	Multiple (N = 526) vs. single jobholders (N = 320)	59.91 vs. 70.52	1.08 (0.85–1.38)	1.09 (0.85–1.39)	1.14 (0.89–1.46)
271–365	Multiple (N = 444) vs. single jobholders (N = 272)	66.03 vs. 74.86	1.04 (0.72–1.49)	1.06 (0.74–1.52)	1.12 (0.78–1.61)

CIP: cumulative incidence proportion, shows the percentages of individuals having returned to work within one year after injury CIP is calculated over full data and evaluated at indicated times; it is not calculated from aggregates shown at left. HR: Hazard ratio; CI: Confidence interval

* Adjusted for MSD, gender, age, occupation, industry, previous claims, and firm size

** Adjusted for variables in model 1, and weekly workdays preceding MSD eligible for compensation benefits

### Likelihood to RTW for MJHs and SJHs, stratified by gender

Table 5 provides proportions of MJHs and SJHs who RTW stratified by gender. Male MJHs were less likely than male SJHs to RTW until 180 days after the first time-loss day, while female MJHs were less likely than female SJHs to RTW only until 60 days after the first time-loss day.

**Table 5:** Likelihood to return to work for multiple jobholders and single jobholders on sickness absence due to an MSD during 1 year follow-up, stratified by gender.

Days after the first time-loss day	Workers not returned to work at end of time frame	CIP %	Crude model (HR (95% CI))	Adjusted model 1* (HR (95% CI))	Adjusted model 2** (HR (95% CI))
**Male** (Multiple (N = 4 279) vs. single jobholders (N = 4 279))					
0–30	Multiple (N = 3 037) vs. single jobholders (N = 2 364)	29.38 vs. 45.03	0.58 (0.54–0.62)	0.58 (0.54–0.62)	0.62 (0.57–0.66)
31–60	Multiple (N = 2 561) vs. single jobholders (N = 1 854)	40.37 vs. 56.95	0.69 (0.61–0.78)	0.68 (0.60–0.77)	0.71 (0.62–0.80)
61–90	Multiple (N = 2 211) vs. single jobholders (N = 1 491)	48.50 vs. 65.34	0.68 (0.58–0.78)	0.67 (0.58–0.77)	0.69 (0.60–0.80)
91–180	Multiple (N = 1 639) vs. single jobholders (N = 983)	61.87 vs. 77.10	0.72 (0.64–0.81)	0.71 (0.63–0.80)	0.74 (0.66–0.84)
181–270	Multiple (N = 1 288) vs. single jobholders (N = 790)	69.94 vs. 81.58	1.10 (0.93–1.32)	1.11 (0.93–1.32)	1.17 (0.98–1.40)
271–365	Multiple (N = 1 115) vs. single jobholders (N = 685)	73.96 vs. 83.99	1.03 (0.80–1.31)	1.05 (0.82–1.33)	1.12 (0.87–1.42)
**Female** (Multiple (N = 4 110) vs. single jobholders (N = 4 110))					
0–30	Multiple (N = 2 660) vs. single jobholders (N = 2 173)	35.86 vs. 47.62	0.67 (0.63–0.72)	0.68 (0.64–0.73)	0.72 (0.67–0.77)
31–60	Multiple (N = 2 052) vs. single jobholders (N = 1 567)	50.29 vs. 62.29	0.78 (0.67–0.87)	0.79 (0.70–0.88)	0.82 (0.73–0.92)
61–90	Multiple (N = 1 587) vs. single jobholders (N = 1 160)	61.78 vs. 72.02	0.88 (0.77–1.00)	0.88 (0.78–1.01)	0.92 (0.81–1.06)
91–180	Multiple (N = 958) vs. single jobholders (N = 685)	76.76 vs. 83.45	0.94 (0.83–1.05)	0.95 (0.84–1.07)	0.98 (0.87–1.11)
181–270	Multiple (N = 710) vs. single jobholders (N = 551)	82.80 vs. 86.62	1.41 (1.14–1.75)	1.45 (1.18–1.80)	1.51 (1.22–1.87)
271–365	Multiple (N = 616) vs. single jobholders (N = 468)	85.04 vs. 88.61	0.85 (0.63–1.15)	0.87 (0.65–1.18)	0.91 (0.68–1.22)

CIP: cumulative incidence proportion, shows the percentages of individuals having returned to work within one year after injury CIP is calculated over full data and evaluated at indicated times; it is not calculated from aggregates shown at left. HR: Hazard ratio; CI: Confidence interval

* Adjusted for MSD, gender, age, occupation, industry, previous claims, and firm size

** Adjusted for variables in model 1, and weekly workdays preceding MSD eligible for compensation benefits

### Likelihood to RTW for MJHs and SJHs, stratified by weekly workdays preceding MSD

Overall, workers who worked six or seven days the week before their MSD (more than the typical workweek) were less likely to RTW than those who worked five or fewer days: 73.05% versus 84.66%. The difference between MJHs and SJHs, whereby MJHs are less likely to RTW than SJHs, was only observed in those who worked five or fewer days (until 180 days after the first time-loss day). Table 6 provides proportions of MJHs and SJHs who RTW stratified by weekly workdays preceding MSD (≤5 versus 6–7).

**Table 6:** Likelihood to return to work for multiple jobholders and single jobholders on sickness absence due to an MSD during 1 year follow-up, stratified by weekly workdays (≤5 versus 6–7).

Days after the first time-loss day	Workers not returned to work at end of time frame	CIP %	Crude model (HR (95% CI))	Adjusted model 1* (HR (95% CI))	Adjusted model 2** (HR (95% CI))
**≤ 5 pre-injury workdays** (Multiple (N = 5 931) vs. single jobholders (N = 7 297))					
0–30	Multiple (N = 3 793) vs. single jobholders (N = 3 688)	36.57 vs. 49.84	0.66 (0.62–0.69)	0.66 (0.63–0.70)	0.66 (0.63–0.70)
31–60	Multiple (N = 3 070) vs. single jobholders (N = 2 753)	48.44 vs. 62.63	0.71 (0.64–0.78)	0.72 (0.65–0.79)	0.69 (0.63–0.77)
61–90	Multiple (N = 2 510) vs. single jobholders (N = 2 095)	58.02 vs. 71.50	0.76 (0.68–0.85)	0.77 (0.68–0.86)	0.75 (0.67–0.84)
91–180	Multiple (N = 1 678) vs. single jobholders (N = 1 272)	71.83 vs. 82.68	0.79 (0.72–0.87)	0.81 (0.74–0.89)	0.80 (0.72–0.88)
181–270	Multiple (N = 1 248) vs. single jobholders (N = 1 013)	79.03 vs. 86.16	1.33 (1.14–1.55)	1.39 (1.19–1.62)	1.38 (1.18–1.61)
271–365	Multiple (N = 1 053) vs. single jobholders (N = 866)	82.28 vs. 88.13	1.09 (0.88–1.36)	1.15 (0.93–1.43)	1.15 (0.93–1.43)
**6–7 pre-injury workdays** (Multiple (N = 2 458) vs. single jobholders (N = 1 092))					
0–30	Multiple (N = 1 904) vs. single jobholders (N = 850)	22.87 vs. 22.62	1.03 (0.89–1.20)	0.99 (0.86–1.15)	0.99 (0.85–1.16)
31–60	Multiple (N = 1 543) vs. single jobholders (N = 668)	37.48 vs. 39.10	0.87 (0.73–1.04)	0.83 (0.69–0.99)	0.84 (0.70–1.00)
61–90	Multiple (N = 1 288) vs. single jobholders (N = 556)	47.74 vs. 49.36	0.97 (0.78–1.22)	0.93 (0.74–1.16)	0.93 (0.74–1.17)
91–180	Multiple (N = 919) vs. single jobholders (N = 398)	62.76 vs. 63.74	1.01 (0.84–1.22)	0.97 (0.80–1.16)	0.97 (0.81–1.18)
181–270	Multiple (N = 750) vs. single jobholders (N = 330)	69.52 vs. 69.96	1.06 (0.80–1.41)	1.04 (0.78–1.38)	1.05 (0.79–1.40)
271–365	Multiple (N = 678) vs. single jobholders (N = 287)	72.41 vs. 73.72	0.74 (0.51–1.09)	0.73 (0.49–1.06)	0.74 (0.50–1.08)

CIP: cumulative incidence proportion, shows the percentages of individuals having returned to work within one year after injury CIP is calculated over full data and evaluated at indicated times; it is not calculated from aggregates shown at left. HR: Hazard ratio; CI: Confidence interval

* Adjusted for MSD, gender, age, occupation, industry, previous claims, and firm size

** Adjusted for variables in model 1, and weekly workdays preceding MSD eligible for compensation benefits

### Likelihood to RTW for MJHs and SJHs, stratified by wage categories

For workers with an annual wage ≤$20 000 (minimum wage), MJHs were less likely to RTW only for the first 30 days after injury compared to SJHs (see Table 7). For workers with an annual wage >$20 000 (minimum wage), MJHs were less likely to RTW the first 180 days after injury compared to SJHs. This is similar to the overall model.

**Table 7:** Likelihood to return to work for multiple jobholders and single jobholders on sickness absence due to an MSD during 1 year follow-up, stratified by wage categories.

Days after the first time-loss day	Workers not returned to work at end of time frame	CIP %	Crude model (HR (95% CI))	Adjusted model 1* (HR (95% CI))	Adjusted model 2** (HR (95% CI))
**< 20 000** (Multiple (N = 657) vs. single jobholders (N = 657))					
0–30	Multiple (N = 428) vs. single jobholders (N = 349)	35.16 vs. 47.03	0.68 (0.57–0.81)	0.69 (0.58–0.82)	0.73 (0.62–0.87)
31–60	Multiple (N = 345) vs. single jobholders (N = 268)	47.64 vs. 59.36	0.82 (0.60–1.11)	0.82 (0.60–1.11)	0.88 (0.65–1.20)
61–90	Multiple (N = 292) vs. single jobholders (N = 226)	56.01 vs. 66.06	0.97 (0.65–1.44)	0.95 (0.64–1.41)	1.03 (0.70–1.54)
91–180	Multiple (N = 213) vs. single jobholders (N = 144)	67.88 vs. 78.23	0.69 (0.50–0.93)	0.66 (0.48–0.90)	0.72 (0.53–0.99)
181–270	Multiple (N = 163) vs. single jobholders (N = 122)	75.49 vs. 81.58	1.66 (1.00–2.73)	1.62 (0.98–2.69)	1.80 (1.09–2.97)
271–365	Multiple (N = 149) vs. single jobholders (N = 111)	77.32 vs. 83.11	0.89 (0.38–2.06)	0.90 (0.39–2.09)	1.00 (0.43–2.33)
**20 000–40 000** (Multiple (N = 2 646) vs. single jobholders (N = 2 646))					
0–30	Multiple (N = 1 766) vs. single jobholders (N = 1 414)	33.95 vs. 46.98	0.64 (0.59–0.70)	0.64 (0.59–0.70)	0.68 (0.62–0.74)
31–60	Multiple (N = 1 431) vs. single jobholders (N = 1 053)	46.09 vs. 60.54	0.69 (0.59–0.80)	0.69 (0.59–0.80)	0.71 (0.61–0.83)
61–90	Multiple (N = 1 169) vs. single jobholders (N = 812)	56.11 vs. 69.39	0.80 (0.67–0.96)	0.80 (0.66–0.95)	0.82 (0.69–0.98)
91–180	Multiple (N = 817) vs. single jobholders (N = 524)	69.15 vs. 80.35	0.79 (0.68–0.93)	0.78 (0.67–0.92)	0.81 (0.70–0.95)
181–270	Multiple (N = 645) vs. single jobholders (N = 439)	75.65 vs. 83.45	1.37 (1.06–1.79)	1.38 (1.07–1.80)	1.44 (1.10–1.87)
271–365	Multiple (N = 569) vs. single jobholders (N = 389)	78.49 vs. 85.30	1.04 (0.73–1.50)	1.05 (0.73–1.50)	1.09 (0.76–1.56)
**40 000–60 000** (Multiple (N = 2 788) vs. single jobholders (N = 2 788))					
0–30	Multiple (N = 1 886) vs. single jobholders (N = 1 513)	32.78 vs. 46.23	0.63 (0.58–0.68)	0.63 (0.58–0.69)	0.67 (0.62–0.73)
31–60	Multiple (N = 1 531) vs. single jobholders (N = 1 128)	45.37 vs. 60.04	0.70 (0.60–0.80)	0.70 (0.60–0.81)	0.72 (0.62–0.84)
61–90	Multiple (N = 1 252) vs. single jobholders (N = 844)	55.38 vs. 70.01	0.71 (0.59–0.83)	0.72 (0.60–0.85)	0.74 (0.63–0.88)
91–180	Multiple (N = 824) vs. single jobholders (N = 530)	70.55 vs. 81.03	0.88 (0.76–1.02)	0.91 (0.78–1.05)	0.93 (0.80–1.07)
181–270	Multiple (N = 637) vs. single jobholders (N = 418)	77.26 vs. 85.04	1.10 (0.87–1.39)	1.16 (0.91–1.46)	1.20 (0.94–1.52)
271–365	Multiple (N = 546) vs. single jobholders (N = 361)	80.42 vs. 87.05	1.04 (0.74–1.45)	1.09 (0.78–1.53)	1.16 (0.83–1.63)
**> 60 000** (Multiple (N = 2 298) vs. single jobholders (N = 2 298))					
0–30	Multiple (N = 1 617) vs. single jobholders (N = 1 262)	29.94 vs. 45.39	0.59 (0.53–0.65)	0.58 (0.52–0.64)	0.61 (0.55–0.67)
31–60	Multiple (N = 1 306) vs. single jobholders (N = 972)	43.39 vs. 57.92	0.82 (0.70–0.96)	0.80 (0.68–0.94)	0.82 (0.70–0.96)
61–90	Multiple (N = 1 085) vs. single jobholders (N = 772)	53.00 vs. 66.75	0.79 (0.65–0.96)	0.78 (0.65–0.95)	0.80 (0.66–0.96)
91–180	Multiple (N = 743) vs. single jobholders (N = 470)	67.89 vs. 79.63	0.79 (0.67–0.91)	0.80 (0.68–0.93)	0.81 (0.69–0.94)
181–270	Multiple (N = 555) vs. single jobholders (N = 364)	75.89 vs. 84.25	1.13 (0.89–1.43)	1.17 (0.92–1.49)	1.21 (0.95–1.53)
271–365	Multiple (N = 467) vs. single jobholders (N = 292)	79.77 vs. 87.29	0.81 (0.59–1.10)	0.86 (0.63–1.18)	0.89 (0.65–1.22)

CIP: cumulative incidence proportion, shows the percentages of individuals having returned to work within one year after injury CIP is calculated over full data and evaluated at indicated times; it is not calculated from aggregates shown at left. HR: Hazard ratio; CI: Confidence interval

* Adjusted for MSD, gender, age, occupation, industry, previous claims, and firm size

** Adjusted for variables in model 1, and weekly workdays preceding MSD eligible for compensation benefits

### Likelihood to RTW for MJHs and SJHs in the validation cohort

Overall, results in the second matched validation cohort were similar to the first matched cohort (S2 Table) with the same conclusions for the effect of MJHs compared to SJHs in general, and by stratifications, until 180 days after the first time-loss day (S3–S7 Tables).

## Discussion

### Main results

To our knowledge, this is the first investigation of the impact of multiple versus single jobholding on the likelihood to RTW for workers with an accepted MSD lost-time claim.

In the **unmatched samples**, we found that on average 6.71% of the workers with an accepted lost-time claim due to a work-related MSD were MJHs. In this sample of workers with a compensation claim for an MSD, this percentage is higher than the 5.50% of MJHs represented in the total labour force in British Columbia [4]. MJHs are more likely women, workers in health and services occupations, workers with a higher income, and with a higher proportion of fractures compared to SJHs. The differences between MJHs and SJHs are consistent with other research literature, showing that MJHs have a higher risk of injury, but are less likely to file a workers’ compensation claim [9–13]. MJHs working conditions may increase the risk of severe injuries, such as fractures, that are due to high workload and related fatigue. However, they may be less likely to submit a workers’ compensation claim for time off work due to precarious employment contracts, which means the proportion of MJHs with work-related injuries would be even higher than presented in the workers’ compensation database used for this study. The higher proportion of women in the MJHs cohort is consistent with labour force information from Statistics Canada [28] and as reported by Tompa et al. [14]. This may be due to women being more likely to have precarious employment contracts that results in having multiple jobs at the same time. Furthermore, more women work in the health and services occupations compared to men, resulting in a higher rate of these occupations in the MJHs cohort [29,30].

The **matched samples** of SJHs and MJHs were balanced on type of MSD, gender, age, wage, occupation and industry. We reduced bias by adjusting for known confounding factors and matching on observed characteristics in the association between multiple jobholding and RTW. Although the risk cannot be eliminated in an observational study design, we reduced residual confounding using random assignment matching.

Overall, MJHs were less likely than SJHs to RTW within the first six months after the first time-loss day of an MSD claim. No differences were found after six months, or MJHs were slightly more likely to RTW than SJHs. MJHs have more precarious job contracts and possibly limited access to support and benefits, which could force MJHs to RTW in the long term for financial reasons. Also, MJHs might be more experienced and resourceful in finding new employment compared to SJHs. It is important to emphasize that 74.69% of the workers with an MSD claim (69.17% of the MJHs and 80.21% of the SJHs) RTW within six months, out of the total 82.83% (79.39% of the MJHs and 86.26% of the SJHs) of workers that RTW in twelve months after injury. So, the difference in time to RTW between MJHs and SJHs within the first six months covers the vast majority of workers. The differences were quite consistent when stratifying by MSD, with the strongest and longest effect shown for fractures. Also, stratifying by serious injury does not explain the difference between MJHs and SJH in likelihood to RTW.

The effect of multiple jobholding on RTW was greater and lasted longer for men than for women. The literature shows that female MJHs are more likely to have two part-time jobs, compared to male MJHs who tend to supplement a full-time job with a second job (explaining their higher income) [8,9]. It might be easier for female MJHs to return to a part-time job, than for male MJHs to return to a full-time job, explaining the observed gender differences in the current study. Furthermore, part-time jobs typically lack the benefits and job security a full-time job may provide, and part-time jobs are more often based on precarious employment contracts for which wages may be lower, creating pressures for (mostly female) MJHs to RTW earlier.

When stratifying the results by workdays preceding MSD, the differences in likelihood to RTW for MJHs versus SJHs remained for workers with five or fewer weekly workdays, but not for those with six or seven workdays. Overall, workers (SJHs and MJHs) who worked six or seven days per week preceding MSD were less likely to RTW, than those who worked five days or less per week. This indicates that working six or seven days per week impedes RTW, regardless of type of jobholding. It reflects the impact of workload on disability and RTW options, and possibly reflects injury severity in workers with a longer than typical five-day workweek [31]. However, only 27% of MJHs and 14% of SJHs worked six or seven days per week. For the majority working five or fewer days per week, MJHs may still have longer hours and higher workload than SJHs, have more precarious employment contracts that decrease access to support like modified RTW programs, or have other underlying factors that explain the reduced likelihood to RTW [14,32].

### Strengths

The major strength of this study is its unique analytical approach, which aims to minimize bias due to different covariates between MJHs and SJHs. Matching by CEM improves balance and achieves more robust inferences than does analysis of an unmatched dataset.

Furthermore, due to the use of comprehensive administrative data in BC, representing 95% of all time-loss claims in the jurisdiction, the large sample enabled the use of more than 90% of the MJHs in the matched cohort, and included a validation cohort to assess the reproducibility of the results from the first matched sample. The similarity in descriptive characteristics between the two cohorts confirm the success of the CEM matching, and the similarity in results verify the validity. The validation cohort guarantees the internal validity of the study methods, while the population based cohort of workers guarantees the external validity of the study results.

### Limitations

Administrative data provides a rich, population-based database with standardized data collection, but data may be subject to misclassification or miscoding, and can lead to information bias. However, cleaning of the data was intensive and only few incomplete claims were excluded from analyses. We were not able to measure other potentially relevant variables, such as: psychosocial (mental aspects of work disability), demographic (race, education, and rural versus urban geographic location), or clinical (e.g. treatment details) factors that could lead to residual confounding [33]. We were also not able to exclude workers with non-work-related fatalities. We estimate non-work-related fatalities to be a very small percentage of workers in our active labour force population during a one year follow-up period. As such, this limitation would be unlikely to change our overall conclusions.

Nevertheless, study results remained consistent after controlling and/or stratifying for key confounders; for example, models that accounted for injury type, such as fractures showed similar results.

Refinement of the workload and RTW measures may help advance our understanding of the relationship between multiple jobholding and RTW in future studies. Although we were able to show a mediating effect of workload on time until RTW, measures of hours worked per week, instead of days, would offer a refinement of the workload measure. In addition, the outcome of time until RTW could be refined to indicate whether MJHs returned to one or more of their jobs. This is important because returning to only one of multiple jobs for a MJH may be an indication of less than full or successful RTW, including reduced earnings and residual disability. This might have impacted our results in such a way that we currently measure that MJHs are returning to at least one of their jobs and the outcome would be full RTW, the likelihood to RTW might be even lower for MJHs compared to SJHs. The ability to measure this would require more sophisticated and sensitive linkage to data such as income tax files, or studies based on sub-sets of the population for which more detailed data can be collected through surveys.

### Interpretation

This study showed the importance of studying MJHs, because of the reduced likelihood for MJHs to RTW in a cohort of injured workers with an accepted MSD lost-time claim compared to SJHs for the first six months after the first time-loss day, representing the majority of workers who is off work after an MSD. Based on the extensive literature search and sensitivity analyses in this study, we suggest that the difference in likelihood to RTW for MJHs compared to SJHs might be caused by precarious employment which challenges RTW in terms of e.g. modified work offerings or being able to return to all types of work and work settings, higher workload, and being less likely to file a workers’ compensation claim. If MJHs are less likely to file a workers’ compensation claim, it may mean that the MJHs cohort has more serious injuries than the SJHs, because they will probably only file a claim for serious injuries. If anything, this suggests that the results would be stronger if all injuries for MJHs were captured. The rise in precarious employment contracts and subsequent MJHs also makes this relevant for further investigation.

### Implications for research and practice

In a previous study, we showed the importance of measuring RTW as a trajectory, and thereby including modified RTW as one of the events in the sickness absence trajectory to explain time to RTW [34]. In this study we were able to show that MJHs in most cases take longer to RTW compared to SJHs, but future studies would benefit from addressing the issue of more refined or detailed measures of RTW, as well as the description of MJHs.

Researchers and policy makers can use the results of this study to identify MJHs with an MSD, especially those with severe injuries and those working six or seven days per week, who may be at risk of delayed RTW; and prioritize interventions and supports for these groups.

## Supporting information

S1 Table Matched variables of single- and multiple jobholders with a time-loss MSD claim between 2010–2014 in the validation cohort. (DOCX)

S2 Table Likelihood to return to work for multiple jobholders and single jobholders on sickness absence due to a MSD during 1 year follow-up in the validation cohort. (DOCX)

S3 Table Likelihood to return to work for multiple jobholders and single jobholders on sickness absence due to a MSD during 1 year follow-up, stratified by MSD in the validation cohort. (DOCX)

S4 Table Likelihood to return to work for multiple jobholders and single jobholders on sickness absence due to a MSD during 1 year follow-up, stratified by serious injury indicator; in the validation cohort. (DOCX)

S5 Table Likelihood to return to work for multiple jobholders and single jobholders on sickness absence due to a MSD during 1 year follow-up, stratified by gender; in the validation cohort. (DOCX)

S6 Table Likelihood to return to work for multiple jobholders and single jobholders on sickness absence due to a MSD during 1 year follow-up, stratified by weekly workdays (≤5 versus 6–7); in the validation cohort. (DOCX)

S7 Table Likelihood to return to work for multiple jobholders and single jobholders on sickness absence due to a MSD during 1 year follow-up, stratified by wage categories; in the validation cohort. (DOCX)
