# Supplementary material for: Return-to-work for multiple jobholders with a work-related musculoskeletal disorder: A population-based, matched cohort in British Columbia
Source: PLoS One. 2018 Apr 3;13(4):e0193618. doi: 10.1371/journal.pone.0193618 (PMC5882128; doi:10.1371/journal.pone.0193618)
Supplement: S1 Table — (DOCX) [file pone.0193618.s001.docx]

**S1 Table. Matched variables of single- and multiple jobholders with a time-loss MSD claim between 2010-2014 in the validation cohort**

|  | **Unmatched sample** | | **Matched sample** | |
| --- | --- | --- | --- | --- |
|  | Single jobholders | Multiple jobholders | Single jobholders | Multiple jobholders |
|  | N= 125 639 (93.3%) | N= 9 029 (6.7%) | N= 8 384 (50%) | N= 8 384 (50%) |
| **Musculoskeletal disorder** |  |  |  |  |
| - Upper Extremity Sprains & Strains | 23 160 (18.4) | 1 865 (20.7) | 1 784 (21.2) | 1 784 (21.2) |
| - Lower Extremity Sprains & Strains | 21 120 (16.8) | 1 610 (17.8) | 1 511 (18.0) | 1 511 (18.0) |
| - Back^b^ Sprains & Strains | 54 379 (43.3) | 3 077 (34.1) | 3 012 (35.9) | 3 012 (35.9) |
| - Upper Extremity Fractures | 6 056 (4.8) | 654 (7.2) | 538 (6.4) | 538 (6.4) |
| - Lower Extremity Fractures | 4 200 (3.3) | 562 (6.2) | 463 (5.5) | 463 (5.5) |
| - Torso^c^ Fractures | 2 477 (2.0) | 216 (2.4) | 161 (1.9) | 161 (1.9) |
| - Dislocation | 2 076 (1.7) | 243 (2.7) | 191 (2.3) | 191 (2.3) |
| - Dorsopathies | 6 300 (5.0) | 388 (4.3) | 347 (4.1) | 347 (4.1) |
| - Rheumatism (Excluding the back) | 5 871 (4.7) | 414 (4.6) | 377 (4.5) | 377 (4.5) |
| **Gender** |  |  |  |  |
| - Male | 78 267 (62.3) | 4 612 (51.1) | 4 297 (51.3) | 4 297 (51.3) |
| - Female | 47 372 (37.7) | 4 417 (48.9) | 4 087 (48.8) | 4 087 (48.8) |
| **Age** |  |  |  |  |
| - 15-24 | 14 580 (11.6) | 900 (9.9) | 790 (9.4) | 790 (9.4) |
| - 25-34 | 26 683 (21.2) | 1 9091 (21.1) | 1 773 (21.2) | 1 773 (21.2) |
| - 35-44 | 28 438 (22.6) | 2 148 (23.8) | 2 005 (23.9) | 2 005 (23.9) |
| - 45-54 | 35 080 (27.9) | 2 767 (30.7) | 2 608 (31.1) | 2 608 (31.1) |
| - 55-64 | 20 858 (16.6) | 1 313 (14.5) | 1 208 (14.4) | 1 208 (14.4) |
| **Wage per annum ($ Canadian)** |  |  |  |  |
| - <$20.000 | 12 431 (9.9) | 773 (8.6) | 658 (7.9) | 658 (7.9) |
| - $20.000-$39.999 | 46 813 (37.3) | 2 789 (30.9) | 2 654 (31.7) | 2 654 (31.7) |
| - $40.000-$59.999 | 37 626 (30.0) | 2 963 (32.8) | 2 773 (33.1) | 2 773 (33.1) |
| - >$59.999 | 28 769 (22.9) | 2 504 (27.7) | 2 299 (27.4) | 2 299 (27.4) |
| **Occupation*** |  |  |  |  |
| - Management | 3 334 (2.7) | 169 (1.9) | 134 (1.6) | 134 (1.6) |
| - Business^d^ | 5 596 (4.5) | 356 (3.9) | 280 (3.3) | 280 (3.3) |
| - Natural sciences^e^ | 2 381 (1.9) | 117 (1.3) | 87 (1.0) | 87 (1.0) |
| - Health | 15 294 (12.2) | 1 659 (18.4) | 1 629 (19.4) | 1 629 (19.4) |
| - Social science^f^ | 5 748 (4.6) | 677 (7.5) | 627 (7.5) | 627 (7.5) |
| - Recreation^g^ | 1 876 (1.5) | 347 (3.8) | 253 (3.0) | 253 (3.0) |
| - Sales, service | 28 549 (22.7) | 2 517 (27.8) | 2 344 (28.0) | 2 344 (28.0) |
| - Trades^h^ | 47 783 (38.0) | 2 393 (26.5) | 2 329 (27.8) | 2 329 (27.8) |
| - Primary industry | 5 431 (4.3) | 305 (3.4) | 257 (3.1) | 257 (3.1) |
| - Manufacturing^i^ | 9 652 (7.7) | 489 (5.4) | 444 (5.3) | 444 (5.3) |
| **Industry*** |  |  |  |  |
| - Primary resources | 4 825 (3.8) | 296 (3 3) | 251 (3.0) | 251 (3.0) |
| - Manufacturing | 15 510 (12.3) | 756 (8.4) | 700 (8.4) | 700 (8.4) |
| - Construction | 17 641 (14.0) | 876 (9.7) | 825 (9.8) | 825 (9.8) |
| - Transportation^j^ | 11 946 (9.5) | 691 (7.7) | 632 (7.5) | 632 (7.5) |
| - Trade | 17 697 (14.1) | 921 (10.2) | 856 (10.2) | 856 (10.2) |
| - Public sector | 5 538 (4.4) | 471 (5.2) | 358 (4.3) | 358 (4.3) |
| - Service sector | 52 482 (41.8) | 5 018 (55.6) | 4 762 (56.8) | 4 762 (56.8) |
| **MULTIVARIATE L1 DISTANCE** |  | 0.394 |  | 0.000 |

^a^ Most responsible firm for the claim of multiple jobholders; ^b^ Back, head, neck, spine and torso; ^c^ Torso, back, neck, spine and head; ^d^ Business, finance and administration; ^e^ Natural and applied sciences, related occupations; ^f^ Social science, education, government, service and religion; ^g^ Recreation, arts, culture and sport; ^h^ Trades, transport, equipment operators and related occupations; ^I^ Manufacturing, processing and utilities; ^j^ Transportation and warehousing
