# Supplementary material for: Return-to-work for multiple jobholders with a work-related musculoskeletal disorder: A population-based, matched cohort in British Columbia
Source: PLoS One. 2018 Apr 3;13(4):e0193618. doi: 10.1371/journal.pone.0193618 (PMC5882128; doi:10.1371/journal.pone.0193618)
Supplement: S2 Table — (DOCX) [file pone.0193618.s002.docx]

**S2 Table. Likelihood to return to work for multiple jobholders and single jobholders on sickness absence due to a MSD during 1 year follow-up in the validation cohort**

| **Days after the first time-loss day** | **Workers not returned to work at end of time frame** (Multiple (N= 8 384) vs. single jobholders (N= 8 384)) | **CIP %** | **Crude model (HR (95% CI))** | **Adjusted model**  **1* (HR (95% CI))** | **Adjusted model**  **2** (HR (95% CI))** |
| --- | --- | --- | --- | --- | --- |
| 0-30 | Multiple (N=5 700) vs. single jobholders (N=4 625) | 32.50 vs. 45.37 | 0.65 (0.62 – 0.68) | 0.65 (0.62 – 0.68) | 0.68 (0.65 – 0.72) |
| 31-60 | Multiple (N=4 616) vs. single jobholders (N=3 435) | 45.16 vs. 59.40 | 0.70 (0.64 – 0.76) | 0.70 (0.64 – 0.76) | 0.72 (0.66 – 0.78) |
| 61-90 | Multiple (N=3 802) vs. single jobholders (N=2 685) | 56.90 vs. 68.27 | 0.79 (0.77 – 0.87) | 0.80 (0.72 – 0.88) | 0.82 (0.74 – 0.90) |
| 91-180 | Multiple (N=2 602) vs. single jobholders (N=1 678) | 69.09 vs. 80.05 | 0.80 (0.74 – 0.87) | 0.81 (0.75 – 0.88) | 0.83 (0.77 – 0.91) |
| 181-270 | Multiple (N=2 003) vs. single jobholders (N=1 341) | 76.15 vs. 84.03 | 1.17 (1.02 – 1.34) | 1.20 (1.04 – 1.37) | 1.25 (1.09 – 1.43) |
| 271-365 | Multiple (N=1 732) vs. single jobholders (N=1 177) | 79.36 vs. 86.00 | 1.40 (0.90 – 1.33) | 1.13 (0.93 – 1.38) | 1.19 (0.98 – 1.44) |

CIP: cumulative incidence proportion, shows the percentages of individuals having returned to work within one year after injury. CIP is calculated over full data and evaluated at indicated times; it is not calculated from aggregates shown at left.. HR: Hazard ratio; CI: Confidence interval; * Adjusted for MSD, gender, age, occupation, industry, previous claims, and firm size; ** Adjusted for variables in model 1, and weekly workdays preceding MSD eligible for compensation benefits
