# Supplementary material for: Return-to-work for multiple jobholders with a work-related musculoskeletal disorder: A population-based, matched cohort in British Columbia
Source: PLoS One. 2018 Apr 3;13(4):e0193618. doi: 10.1371/journal.pone.0193618 (PMC5882128; doi:10.1371/journal.pone.0193618)
Supplement: S3 Table — (DOCX) [file pone.0193618.s003.docx]

**S3 Table. Likelihood to return to work for multiple jobholders and single jobholders on sickness absence due to a MSD during 1 year follow-up, stratified by MSD in the validation cohort**

| **Days after the first time-loss day** | **Workers not returned to work at end of time frame** | **CIP %** | **Crude model (HR (95% CI))** | **Adjusted model**  **1* (HR (95% CI))** | **Adjusted model**  **2** (HR (95% CI))** |
| --- | --- | --- | --- | --- | --- |
| **Upper extremity sprains & strains** (Multiple (N=1 784) vs. single jobholders (N=1 784)) | | | | | |
| 0-30 | Multiple (N=1 279) vs. single jobholders (N=991) | 28.81 vs. 44.79 | 0.57 (0.51 – 0.64) | 0.57 (0.51 – 0.64) | 0.61 (0.54 – 0.68) |
| 31-60 | Multiple (N=1 051) vs. single jobholders (N=748) | 41.26 vs. 56.58 | 0.67 (0.56 – 0.80) | 0.67 (0.56 – 0.81) | 0.69 (0.58 – 0.83) |
| 61-90 | Multiple (N=885) vs. single jobholders (N=606) | 50.73 vs. 66.31 | 0.84 (0.67 – 1.05) | 0.85 (0.68 – 1.07) | 0.89 (0.71 – 1.11) |
| 91-180 | Multiple (N=631) vs. single jobholders (N=398) | 64.69 vs. 77.80 | 0.78 (0.65 – 0.94) | 0.80 (0.66 – 0.95) | 0.81 (0.68 – 0.98) |
| 181-270 | Multiple (N=508) vs. single jobholders (N=329) | 71.58 vs. 81.61 | 1.16 (0.86 – 1.56) | 1.18 (0.88 – 1.59) | 1.24 (0.92 – 1.68) |
| 271-365 | Multiple (N=442) vs. single jobholders (N=290) | 75.22 vs. 83.80 | 1.08 (0.72 – 1.60) | 1.11 (0.74 – 1.65) | 1.18 (0.79 – 1.76) |
| **Lower extremity sprains & strains** (Multiple (N=1 511) vs. single jobholders (N=1 511)) | | | | | |
| 0-30 | Multiple (N=920) vs. single jobholders (N=695) | 39.54 vs. 54.53 | 0.63 (0.57 – 0.70) | 0.64 (0.57 – 0.71) | 0.67 (0.60 – 0.74) |
| 31-60 | Multiple (N=743) vs. single jobholders (N=500) | 50.99 vs. 67.11 | 0.65 (0.53 – 0.80) | 0.66 (0.54 – 0.81) | 0.67 (0.54 – 0.82) |
| 61-90 | Multiple (N=620) vs. single jobholders (N=408) | 59.07 vs. 73.40 | 0.85 (0.65 – 1.12) | 0.88 (0.67 – 1.14) | 0.90 (0.69 – 1.18) |
| 91-180 | Multiple (N=450) vs. single jobholders (N=275) | 70.33 vs. 82.00 | 0.81 (0.65 – 1.03) | 0.84 (0.67 – 1.05) | 0.86 (0.68 – 1.08) |
| 181-270 | Multiple (N=325) vs. single jobholders (N=202) | 78.54 vs. 86.70 | 1.09 (0.81 – 1.45) | 1.11 (0.83 – 1.49) | 1.15 (0.86 – 1.54) |
| 271-365 | Multiple (N=268) vs. single jobholders (N=168) | 82.32 vs. 88.95 | 1.05 (0.68 – 1.60) | 1.07 (0.70 – 1.65) | 1.10 (0.72 – 1.69) |
| **Back sprains & strains** (Multiple (N=3 012) vs. single jobholders (N= 3 012)) | | | | | |
| 0-30 | Multiple (N=1 759) vs. single jobholders (N=1 426) | 42.18 vs.53.25 | 0.71 (0.66 – 0.76) | 0.72 (0.67 – 0.77) | 0.76 (0.70 – 0.81) |
| 31-60 | Multiple (N=1 259) vs. single jobholders (N=952) | 58.49 vs. 68.69 | 0.82 (0.73 – 0.94) | 0.83 (0.73 – 0.95) | 0.86 (0.76 – 0.98) |
| 61-90 | Multiple (N=951) vs. single jobholders (N=661) | 68.75 vs. 78.39 | 0.77 (0.66 – 0.90) | 0.78 (0.67 – 0.92) | 0.80 (0.68 – 0.94) |
| 91-180 | Multiple (N=581) vs. single jobholders (N=395) | 80.74 vs. 86.92 | 0.96 (0.82 – 1.12) | 0.99 (0.84 – 1.16) | 1.01 (0.86 – 1.18) |
| 181-270 | Multiple (N=475) vs. single jobholders (N=342) | 84.26 vs. 88.68 | 1.39 (1.00– 1.94) | 1.46 (1.05 – 2.04) | 1.52 (1.09 – 2.11) |
| 271-365 | Multiple (N=440) vs. single jobholders (N=316) | 85.39 vs. 89.51 | 0.98 (0.58 – 1.64) | 1.03 (0.62 – 1.73) | 1.07 (0.64 – 1.80) |
| **Upper extremity fractures** (Multiple (N=538) vs. single jobholders (N=538)) | | | | | |
| 0-30 | Multiple (N=462) vs. single jobholders (N=418) | 14.68 vs. 22.49 | 0.63 (0.47 – 0.83) | 0.64 (0.48 – 0.85) | 0.67 (0.51 – 0.88) |
| 31-60 | Multiple (N=409) vs. single jobholders (N=332) | 24.16 vs. 38.48 | 0.50 (0.36 – 0.71) | 0.50 (0.36 – 0.71) | 0.52 (0.37 – 0.74) |
| 61-90 | Multiple (N=334) vs. single jobholders (N=261) | 38.29 vs. 51.67 | 0.84 (0.61 – 1.16) | 0.84 (0.61 – 1.16) | 0.88 (0.64 – 1.22) |
| 91-180 | Multiple (N=197) vs. single jobholders (N=128) | 63.57 vs. 76.39 | 0.72 (0.56 – 0.91) | 0.72 (0.56 – 0.91) | 0.75 (0.59 – 0.96) |
| 181-270 | Multiple (N=147) vs. single jobholders (N=98) | 72.86 vs. 81.97 | 1.07 (0.68 – 1.69) | 1.07 (0.68 – 1.69) | 1.17 (0.75 – 1.86) |
| 271-365 | Multiple (N=121) vs. single jobholders (N=87) | 77.51 vs. 84.01 | 1.59 (0.78 – 3.23) | 1.60 (0.78 – 3.25) | 1.74 (0.86 – 3.55) |
| **Lower extremity fractures** (Multiple (N=463) vs. single jobholders (N=463)) | | | | | |
| 0-30 | Multiple (N=436) vs. single jobholders (N=387) | 6.48 vs. 16.85 | 0.36 (0.24 – 0.55) | 0.36 (0.24 – 0.55) | 0.38 (0.25 – 0.58) |
| 31-60 | Multiple (N=412) vs. single jobholders (N=329) | 11.45 vs. 29.59 | 0.33 (0.20 – 0.53) | 0.32 (0.20 – 0.51) | 0.34 (0.21 – 0.55) |
| 61-90 | Multiple (N=359) vs. single jobholders (N=263) | 22.68 vs. 43.41 | 0.62 (0.43 – 0.89) | 0.59 (0.41 – 0.85) | 0.62 (0.43 – 0.90) |
| 91-180 | Multiple (N=263) vs. single jobholders (N=175) | 44.06 vs. 62.42 | 0.78 (0.58 – 1.04) | 0.74 (0.55 – 0.98) | 0.78 (0.58 – 1.04) |
| 181-270 | Multiple (N=195) vs. single jobholders (N=129) | 58.10 vs. 72.35 | 0.96 (0.66 – 1.39) | 0.92 (0.63 – 1.35) | 0.97 (0.66 – 1.43) |
| 271-365 | Multiple (N=171) vs. single jobholders (N=110) | 63.07 vs. 76.24 | 0.81 (0.44 – 1.50) | 0.79 (0.42 – 1.45) | 0.85 (0.46 – 1.58) |
| **Torso fractures** (Multiple (N=161) vs. single jobholders (N=161)) | | | | | |
| 0-30 | Multiple (N=139) vs. single jobholders (N=124) | 14.29 vs. 24.84 | 0.54 (0.32 – 0.90) | 0.55 (0.33 – 0.93) | 0.62 (0.37 – 1.06) |
| 31-60 | Multiple (N=125) vs. single jobholders (N=94) | 22.98 vs. 43.48 | 0.37 (0.20 – 0.71) | 0.38 (0.20 – 0.73) | 0.39 (0.21 – 0.75) |
| 61-90 | Multiple (N=118) vs. single jobholders (N=77) | 27.33 vs. 52.80 | 0.32 (0.13 – 0.78) | 0.34 (0.14 – 0.84) | 0.35 (0.14 – 0.87) |
| 91-180 | Multiple (N=99) vs. single jobholders (N=49) | 39.13 vs. 70.19 | 0.36 (0.21 – 0.66) | 0.36 (0.20 – 0.64) | 0.33 (0.18 – 0.60) |
| 181-270 | Multiple (N=78) vs. single jobholders (N=44) | 52.17 vs. 73.29 | 2.13 (0.81 – 5.66) | 1.96 (0.74 – 5.23) | 1.75 (0.65 – 4.68) |
| 271-365 | Multiple (N=66) vs. single jobholders (N=41) | 59.01 vs. 74.53 | 3.23 (0.72 – 14.57) | 3.01 (0.66 – 13.65) | 2.71 (0.59 – 12.31) |
| **Dislocations** (Multiple (N=191) vs. single jobholders (N=191)) | | | | | |
| 0-30 | Multiple (N=167) vs. single jobholders (N=149) | 13.61 vs. 22.51 | 0.58 (0.36 – 0.94) | 0.57 (0.35 – 0.93) | 0.52 (0.31 – 0.86) |
| 31-60 | Multiple (N=142) vs. single jobholders (N=134) | 26.18 vs. 30.37 | 1.44 (0.76 – 2.76) | 1.41 (0.74 – 2.71) | 1.39 (0.73 – 2.67) |
| 61-90 | Multiple (N=133) vs. single jobholders (N=125) | 30.89 vs. 35.60 | 0.84 (0.34 – 2.08) | 0.83 (0.34 – 2.06) | 0.75 (0.29 – 1.90) |
| 91-180 | Multiple (N=101) vs. single jobholders (N=90) | 47.64 vs. 53.40 | 0.84 (0.52 – 1.36) | 0.84 (0.52 – 1.37) | 0.87 (0.53 – 1.43) |
| 181-270 | Multiple (N=78) vs. single jobholders (N=61) | 59.69 vs. 68.59 | 0.66 (0.38 – 1.15) | 0.64 (0.37 – 1.12) | 0.68 (0.39 – 1.19) |
| 271-365 | Multiple (N=58) vs. single jobholders (N=42) | 70.16 vs. 78.01 | 0.79 (0.41 – 1.50) | 0.74 (0.39 – 1.42) | 0.77 (0.40 – 1.47) |
| **Dorsopathies** (Multiple (N=347) vs. single jobholders (N=347)) | | | | | |
| 0-30 | Multiple (N=246) vs. single jobholders (N=191) | 29.68 vs. 46.11 | 0.59 (0.46 – 0.75) | 0.59 (0.46 – 0.76) | 0.65 (0.51 – 0.85) |
| 31-60 | Multiple (N=208) vs. single jobholders (N=152) | 40.35 vs. 56.48 | 0.78 (0.49 – 1.23) | 0.79 (0.50 – 1.26) | 0.81 (0.51 – 1.29) |
| 61-90 | Multiple (N=178) vs. single jobholders (N=131) | 49.57 vs. 62.54 | 1.11 (0.65 – 1.94) | 1.16 (0.67 – 2.01) | 1.11 (0.64 – 1.93) |
| 91-180 | Multiple (N=136) vs. single jobholders (N=81) | 61.38 vs. 76.95 | 0.54 (0.36 – 0.82) | 0.56 (0.37 – 0.84) | 0.52 (0.34 – 0.80) |
| 181-270 | Multiple (N=101) vs. single jobholders (N=66) | 71.18 vs. 81.27 | 1.40 (0.77 – 2.59) | 1.46 (0.79 – 2.69) | 1.37 (0.74 – 2.54) |
| 271-365 | Multiple (N=85) vs. single jobholders (N=55) | 75.50 vs. 84.15 | 1.00 (0.45 – 2.24) | 1.08 (0.49 – 2.43) | 1.04 (0.46 – 2.31) |
| **Rheumatism (excluding the back)** (Multiple (N=377) vs. single jobholders (N=377)) | | | | | |
| 0-30 | Multiple (N=298) vs. single jobholders (N=244) | 21.75 vs. 35.81 | 0.55 (0.42 – 0.72) | 0.55 (0.42 – 0.73) | 0.59 (0.44 – 0.78) |
| 31-60 | Multiple (N=270) vs. single jobholders (N=194) | 28.65 vs. 48.81 | 0.41 (0.26 – 0.66) | 0.41 (0.26 – 0.67) | 0.43 (0.27 – 0.70) |
| 61-90 | Multiple (N=229) vs. single jobholders (N=157) | 39.52 vs. 58.89 | 0.77 (0.49 – 1.19) | 0.78 (0.50 – 1.21) | 0.78 (0.50 – 1.22) |
| 91-180 | Multiple (N=147) vs. single jobholders (N=94) | 61.27 vs. 75.33 | 0.84 (0.61 – 1.18) | 0.86 (0.62 – 1.20) | 0.88 (0.63 – 1.23) |
| 181-270 | Multiple (N=101) vs. single jobholders (N=77) | 73.47 vs. 79.84 | 1.85 (1.06 – 3.23) | 1.91 (1.09 – 3.33) | 1.97 (1.12 – 3.44) |
| 271-365 | Multiple (N=81) vs. single jobholders (N=68) | 78.51 vs. 81.96 | 1.91 (0.83 – 4.37) | 1.92 (0.84 – 4.39) | 2.00 (0.87 – 4.57) |

CIP: cumulative incidence proportion, shows the percentages of individuals having returned to work within one year after injury CIP is calculated over full data and evaluated at indicated times; it is not calculated from aggregates shown at left.. HR: Hazard ratio; CI: Confidence interval; * Adjusted for MSD, gender, age, occupation, industry, previous claims, and firm size; ** Adjusted for variables in model 1, and weekly workdays preceding MSD eligible for compensation benefits
