# Supplementary material for: Return-to-work for multiple jobholders with a work-related musculoskeletal disorder: A population-based, matched cohort in British Columbia
Source: PLoS One. 2018 Apr 3;13(4):e0193618. doi: 10.1371/journal.pone.0193618 (PMC5882128; doi:10.1371/journal.pone.0193618)
Supplement: S4 Table — (DOCX) [file pone.0193618.s004.docx]

**S4 Table. Likelihood to return to work for multiple jobholders and single jobholders on sickness absence due to a MSD during 1 year follow-up, stratified by serious injury indicator; in the validation cohort.**

| **Days after the first time-loss day** | **Workers not returned to work at end of time frame** | **CIP %** | **Crude model  (HR (95% CI))** | **Adjusted model**  **1* (HR (95% CI))** | **Adjusted model**  **2** (HR (95% CI))** |
| --- | --- | --- | --- | --- | --- |
| **No serious injury** (Multiple (N=7 065) vs. single jobholders (N=7 271)) | | | | | |
| 0-30 | Multiple (N=4 474) vs. single jobholders (N=3 633) | 37.24 vs. 50.61 | 0.66 (0.62 – 0.69) | 0.67 (0.63 – 0.70) | 0.70 (0.66 – 0.74) |
| 31-60 | Multiple (N=3 478) vs. single jobholders (N=2 550) | 51.00 vs. 65.26 | 0.70 (0.64 – 0.77) | 0.72 (0.66 – 0.78) | 0.73 (0.67 – 0.80) |
| 61-90 | Multiple (N=2 80) vs. single jobholders (N=1 957) | 60.53 vs. 73.41 | 0.81 (0.72 – 0.90) | 0.83 (0.74 – 0.92) | 0.84 (0.75 – 0.94) |
| 91-180 | Multiple (N=1 895) vs. single jobholders (N= 1 242) | 73.25 vs. 82.97 | 0.86 (0.78 – 0.95) | 0.88 (0.80 – 0.97) | 0.90 (0.81 – 0.99) |
| 181-270 | Multiple (N=1 476) vs. single jobholders (N= 1 013) | 79.13 vs. 86.08 | 1.23 (1.05 – 1.45) | 1.28 (1.09 – 1.50) | 1.32 (1.12 – 1.55) |
| 271-365 | Multiple (N=1 287) vs. single jobholders (N=910) | 81.81 vs. 87.64 | 1.16 (0.92 – 1.46) | 1.20 (0.95 – 1.52) | 1.25 (0.99 – 1.57) |
| **Serious injury** (Multiple (N=1 319) vs. single jobholders (N=1 113)) | | | | | |
| 0-30 | Multiple (N=1 226) vs. single jobholders (N=992) | 7.13 vs. 11.14 | 0.63 (0.48 – 0.82) | 0.64 (0.49 – 0.84) | 0.66 (0.50 – 0.86) |
| 31-60 | Multiple (N=1 138) vs. single jobholders (N=885) | 13.87 vs. 21.11 | 0.63 (0.48 – 0.83) | 0.64 (0.49 – 0.84) | 0.67 (0.51 – 0.89) |
| 61-90 | Multiple (N=995) vs. single jobholders (N=728) | 26.79 vs. 34.68 | 0.71 (0.57 – 0.89) | 0.72 (0.57 – 0.91) | 0.76 (0.59 – 0.94) |
| 91-180 | Multiple (N=707) vs. single jobholders (N=436) | 46.78 vs. 60.92 | 0.66 (0.56 – 0.78) | 0.66 (0.56 – 0.78) | 0.70 (0.59 – 0.82) |
| 181-270 | Multiple (N=527) vs. single jobholders (N=328) | 60.20 vs. 70.62 | 1.02 (0.80 – 1.29) | 1.03 (0.81 – 1.32) | 1.10 (0.87 – 1.40) |
| 271-365 | Multiple (N=445) vs. single jobholders (N=276) | 66.26 vs. 75.29 | 0.95 (0.67 – 1.35) | 0.98 (0.69 – 1.39) | 1.05 (0.74 – 1.96) |

CIP: cumulative incidence proportion, shows the percentages of individuals having returned to work within one year after injury CIP is calculated over full data and evaluated at indicated times; it is not calculated from aggregates shown at left.. HR: Hazard ratio; CI: Confidence interval; * Adjusted for MSD, gender, age, occupation, industry, previous claims, and firm size; ** Adjusted for variables in model 1, and weekly workdays preceding MSD eligible for compensation benefits
