# Supplementary material for: Return-to-work for multiple jobholders with a work-related musculoskeletal disorder: A population-based, matched cohort in British Columbia
Source: PLoS One. 2018 Apr 3;13(4):e0193618. doi: 10.1371/journal.pone.0193618 (PMC5882128; doi:10.1371/journal.pone.0193618)
Supplement: S5 Table — (DOCX) [file pone.0193618.s005.docx]

**S5 Table. Likelihood to return to work for multiple jobholders and single jobholders on sickness absence due to a MSD during 1 year follow-up, stratified by gender; in the validation cohort**

| **Days after the first time-loss day** | **Workers not returned to work at end of time frame** | **CIP %** | **Crude model (HR (95% CI))** | **Adjusted model**  **1* (HR (95% CI))** | **Adjusted model**  **2** (HR (95% CI))** |
| --- | --- | --- | --- | --- | --- |
| **Male** (Multiple (N=4 662) vs. single jobholders (N=4 662)) | | | | | |
| 0-30 | Multiple (N=2 947) vs. single jobholders (N=2 591) | 29.31 vs. 43.96 | 0.60 (0.56 – 0.64) | 0.60 (0.56 – 0.64) | 0.63 (0.58 – 0.67) |
| 31-60 | Multiple (N=2 484) vs. single jobholders (N=2 019) | 40.35 vs. 56.97 | 0.64 (0.57 – 0.72) | 0.63 (0.56 – 0.71) | 0.65 (0.57 – 0.73) |
| 61-90 | Multiple (N=2 148) vs. single jobholders (N=1 591) | 48.43 vs. 64.95 | 0.71 (0.60 – 0.82) | 0.70 (0.60 – 0.81) | 0.72 (0.62 – 0.83) |
| 91-180 | Multiple (N=1 618) vs. single jobholders (N=1 060) | 61.96 vs. 76.66 | 0.74 (0.66 – 0.83) | 0.72 (0.64 – 0.82) | 0.75 (0.66 – 0.84) |
| 181-270 | Multiple (N=1 289) vs. single jobholders (N=843) | 69.94 vs. 81.34 | 1.07 (0.89 – 1.27) | 1.05 (0.88 – 1.24) | 1.08 (0.91 – 1.29) |
| 271-365 | Multiple (N=1 113) vs. single jobholders (N=719) | 74.06 vs. 839.99 | 0.97 (0.76 – 1.22) | 0.96 (0.76 – 1.22) | 0.99 (0.79 – 1.26) |
| **Female** (Multiple (N=4 337) vs. single jobholders (N=4 337)) | | | | | |
| 0-30 | Multiple (N=2 596) vs. single jobholders (N=2 318) | 35.85 vs. 46.86 | 0.69 (0.64 – 0.74) | 0.70 (0.65 – 0.74) | 0.74 (0.69 – 0.79) |
| 31-60 | Multiple (N=1 983) vs. single jobholders (N=1 688) | 50.21 vs. 61.95 | 0.76 (0.68 – 0.85) | 0.87 (0.68 – 0.86) | 0.79 (0.71 – 0.89) |
| 61-90 | Multiple (N=1 527) vs. single jobholders (N=1 241) | 61.71 vs. 71.76 | 0.88 (0.77 – 1.01) | 0.89 (0.78 – 1.03) | 0.92 (0.80 – 1.05) |
| 91-180 | Multiple (N=939) vs. single jobholders (N=747) | 76.58 vs. 83.61 | 0.88 (0.79 – 1.00) | 0.91 (0.81 – 1.03) | 0.94 (0.83 – 1.06) |
| 181-270 | Multiple (N=707) vs. single jobholders (N=600) | 82.68 vs. 86.86 | 1.35 (1.09 – 1.66) | 1.42 (1.15 – 1.76) | 1.49 (1.51 – 1.84) |
| 271-365 | Multiple (N=614) vs. single jobholders (N=535) | 84.93 vs. 88.11 | 1.38 (0.98 – 1.94) | 1.45 (1.03 – 2.04) | 1.53 (1.08 – 2.15) |

CIP: cumulative incidence proportion, shows the percentages of individuals having returned to work within one year after injury CIP is calculated over full data and evaluated at indicated times; it is not calculated from aggregates shown at left.. HR: Hazard ratio; CI: Confidence interval; * Adjusted for MSD, gender, age, occupation, industry, previous claims, and firm size; ** Adjusted for variables in model 1, and weekly workdays preceding MSD eligible for compensation benefits
