# Supplementary material for: Return-to-work for multiple jobholders with a work-related musculoskeletal disorder: A population-based, matched cohort in British Columbia
Source: PLoS One. 2018 Apr 3;13(4):e0193618. doi: 10.1371/journal.pone.0193618 (PMC5882128; doi:10.1371/journal.pone.0193618)
Supplement: S6 Table — (DOCX) [file pone.0193618.s006.docx]

**S6 Table. Likelihood to return to work for multiple jobholders and single jobholders on sickness absence due to a MSD during 1 year follow-up, stratified by weekly workdays (≤5 versus 6-7); in the validation cohort**

| **Days after the first time-loss day** | **Workers not returned to work at end of time frame** | **CIP %** | **Crude model  (HR (95% CI))** | **Adjusted model**  **1* (HR (95% CI))** | **Adjusted model**  **2** (HR (95% CI))** |
| --- | --- | --- | --- | --- | --- |
| **≤ 5 pre-injury workdays** (Multiple (N=6 450) vs. single jobholders (N=7 785)) | | | | | |
| 0-30 | Multiple (N=3 671) vs. single jobholders (N=3 952) | 36.51 vs. 48.97 | 0.68 (0.64 – 0.71) | 0.68 (0.65 – 0.72) | 0.68 (0.64 – 0.72) |
| 31-60 | Multiple (N=2 957) vs. single jobholders (N=2 933) | 48.36 vs. 62.46 | 0.67 (0.61 – 0.74) | 0.68 (0.62 – 0.75) | 0.66 (0.60 – 0.73) |
| 61-90 | Multiple (N=2 420) vs. single jobholders (N=2 198) | 57.86 vs. 70.86 | 0.80 (0.71 – 0.90) | 0.81 (0.72 – 0.91) | 0.79 (0.71 – 0.89) |
| 91-180 | Multiple (N=1 665) vs. single jobholders (N=1 358) | 71.65 vs. 82.22 | 0.79 (0.72 – 0.87) | 0.81 (0.74 – 0.89) | 0.79 (0.72 – 0.87) |
| 181-270 | Multiple (N=1 255) vs. single jobholders (N=1 067) | 78.91 vs. 85.82 | 1.32 (1.13 – 1.54) | 1.36 (0.17 – 0.59) | 1.35 (1.16 – 1.58) |
| 271-365 | Multiple (N=1 062) vs. single jobholders (N=929) | 82.16 vs. 87.66 | 1.20 (0.96 – 1.50) | 1.26 (1.00 – 1.57) | 1.25 (1.00 – 1.56) |
| **6 – 7 pre-injury workdays** (Multiple (N=2529) vs. single jobholders (N=1 212)) | | | | | |
| 0-30 | Multiple (N=1 872) vs. single jobholders (N=262) | 22.71 vs. 22.40 | 1.03 (0.89 – 1.19) | 0.99 (0.86 – 1.15) | 0.99 (0.85 – 1.16) |
| 31-60 | Multiple (N=1 510) vs. single jobholders (N=182) | 37.33 vs. 39.86 | 0.82 (0.69 – 0.98) | 0.80 (0.67 – 0.95) | 0.81 (0.68 – 0.96) |
| 61-90 | Multiple (N=1 255) vs. single jobholders (N=140) | 47.68 vs. 51.76 | 0.82 (0.67 – 1.01) | 0.80 (0.65 – 0.90) | 0.80 (0.65 – 0.99) |
| 91-180 | Multiple (N=902) vs. single jobholders (N=183) | 62.83 vs. 66.14 | 0.95 (0.79 – 1.14) | 0.92 (0.77 – 1.11) | 0.92 (0.77 – 1.11) |
| 181-270 | Multiple (N=741) vs. single jobholders (N=75) | 69.40 vs. 72.57 | 0.92 (0.70 – 1.21) | 0.90 (0.68 – 1.19) | 0.91 (0.69 – 1.20) |
| 271-365 | Multiple (N=665) vs. single jobholders (N=50) | 72.53 vs. 75.40 | 0.99 (0.66 – 1.50) | 0.99 (0.65 – 1.49) | 0.99 (0.66 – 1.50) |

CIP: cumulative incidence proportion, shows the percentages of individuals having returned to work within one year after injury CIP is calculated over full data and evaluated at indicated times; it is not calculated from aggregates shown at left.. HR: Hazard ratio; CI: Confidence interval; * Adjusted for MSD, gender, age, occupation, industry, previous claims, and firm size; ** Adjusted for variables in model 1, and weekly workdays preceding MSD eligible for compensation benefits
