# Supplementary material for: Return-to-work for multiple jobholders with a work-related musculoskeletal disorder: A population-based, matched cohort in British Columbia
Source: PLoS One. 2018 Apr 3;13(4):e0193618. doi: 10.1371/journal.pone.0193618 (PMC5882128; doi:10.1371/journal.pone.0193618)
Supplement: S7 Table — (DOCX) [file pone.0193618.s007.docx]

**S7 Table. Likelihood to return to work for multiple jobholders and single jobholders on sickness absence due to a MSD during 1 year follow-up, stratified by wage categories; in the validation cohort**

| **Days after the first time-loss day** | **Workers not returned to work at end of time frame** | **CIP %** | **Crude model  (HR (95% CI))** | **Adjusted model**  **1* (HR (95% CI))** | **Adjusted model**  **2** (HR (95% CI))** |
| --- | --- | --- | --- | --- | --- |
| **< 20 000** (Multiple (N=658) vs. single jobholders (N=658)) | | | | | |
| 0-30 | Multiple (N=427) vs. single jobholders (N=376) | 35.56 vs. 44.07 | 0.75 (0.63 – 0.89) | 0.76 (0.64 – 0.90) | 0.80 (0.67 – 0.95) |
| 31-60 | Multiple (N=344) vs. single jobholders (N=290) | 47.87 vs. 56.23 | 0.86 (0.63 – 1.17) | 0.87 (0.64 – 1.19) | 0.92 (0.68 – 1.26) |
| 61-90 | Multiple (N=292) vs. single jobholders (N=238) | 55.93 vs. 64.29 | 0.82 (0.56 – 1.21) | 0.84 (0.58 – 1.23) | 0.89 (0.30 – 1.30) |
| 91-180 | Multiple (N=214) vs. single jobholders (N=142) | 67.78 vs. 78.57 | 0.59 (0.44 – 0.80) | 0.60 (0.45 – 0.82) | 0.64 (0.47 – 0.86) |
| 181-270 | Multiple (N=166) vs. single jobholders (N=122) | 75.08 vs. 81.61 | 1.69 (1.00 – 2.86) | 1.77 (1.05 – 2.98) | 1.86 (1.10 – 3.14) |
| 271-365 | Multiple (N=151) vs. single jobholders (N=117) | 77.05 vs. 82.22 | 2.42 (0.79 – 7.43) | 2.59 (0.84 – 7.95) | 2.74 (0.89 – 8.42) |
| **20 000 – 40 000** (Multiple (N=2 654) vs. single jobholders (N=2 654)) | | | | | |
| 0-30 | Multiple (N=1 770) vs. single jobholders (N=1 423) | 34.01 vs. 46.80 | 0.65 (0.60 – 0.71) | 0.65 (0.60 – 0.71) | 0.67 (0.62 – 0.73) |
| 31-60 | Multiple (N=1 439) vs. single jobholders (N=1 068) | 45.93 vs. 60.17 | 0.69 (0.59 – 0.80) | 0.69 (0.59 – 0.80) | 0.70 (0.60 – 0.82) |
| 61-90 | Multiple (N=1 172) vs. single jobholders (N=811) | 56.11 vs. 69.82 | 0.75 (0.63 – 0.89) | 0.75 (0.63 – 0.89) | 0.76 (0.64 – 0.90) |
| 91-180 | Multiple (N=815) vs. single jobholders (N=510) | 69.34 vs. 80.86 | 0.78 (0.67 – 0.92) | 0.78 (0.67 – 0.91) | 0.79 (0.67 – 0.92) |
| 181-270 | Multiple (N=643) vs. single jobholders (N=409) | 75.79 vs. 84.63 | 1.07 (0.84 – 1.37) | 1.07 (0.83 – 1.37) | 1.10 (0.86 – 1.41) |
| 271-365 | Multiple (N=569) vs. single jobholders (N=375) | 78.54 vs. 85.91 | 1.38 (0.92 – 2.07) | 1.37 (0.91 – 2.05) | 1.42 (0.94 – 2.13) |
| **40 000 – 60 000** (Multiple (N=2 773) vs. single jobholders (N=2 773)) | | | | | |
| 0-30 | Multiple (N=1 885) vs. single jobholders (N=1 529) | 32.46 vs. 45.33 | 0.65 (0.60 – 0.71) | 0.66 (0.60 – 0.71) | 0.69 (0.63 – 0.75) |
| 31-60 | Multiple (N=1 529) vs. single jobholders (N=1 1102) | 45.15 vs. 60.55 | 0.64 (0.55 – 0.73) | 0.65 (0.56 – 0.75) | 0.66 (0.58 – 0.77) |
| 61-90 | Multiple (N=1 250) vs. single jobholders (N=842) | 55.17 vs. 70.00 | 0.74 (0.62 – 0.87) | 0.76 (0.64 – 0.90) | 0.78 (0.66 – 0.92) |
| 91-180 | Multiple (N=825) vs. single jobholders (N=516) | 70.36 vs. 81.50 | 0.83 (0.72 – 0.96) | 0.88 (0.76 – 1.01) | 0.89 (0.77 – 1.03) |
| 181-270 | Multiple (N=637) vs. single jobholders (N=417) | 77.10 vs. 85.00 | 1.24 (0.97 – 1.59) | 1.34 (1.04 – 1.71) | 1.37 (1.07 – 1.76) |
| 271-365 | Multiple (N=542) vs. single jobholders (N=362) | 80.45 vs. 86.98 | 1.11 (0.79 – 1.55) | 1.21 (0.86 – 1.69) | 1.26 (0.91 – 1.77) |
| **> 60 000** (Multiple (N=2 299) vs. single jobholders (N=2 299)) | | | | | |
| 0-30 | Multiple (N=1 618) vs. single jobholders (N=1 297) | 29.93 vs. 44.15 | 0.61 (0.55 – 0.67) | 0.59 (0.54 – 0.66) | 0.63 (0.58 – 0.70) |
| 31-60 | Multiple (N=1 304) vs. single jobholders (N=975) | 43.50 vs. 58.03 | 0.75 (0.64 – 0.88) | 0.73 (0.62 – 0.85) | 0.75 (0.64 – 0.88) |
| 61-90 | Multiple (N=1 088) vs. single jobholders (N=797) | 52.89 vs. 65.55 | 0.92 (0.75 – 1.12) | 0.89 (0.73 – 1.09) | 0.92 (0.75 – 0.12) |
| 91-180 | Multiple (N=748) vs. single jobholders (N=512) | 67.64 vs. 77.77 | 0.85 (0.73 – 0.99) | 0.84 (0.72 – 0.99) | 0.87 (0.74 – 1.02) |
| 181-270 | Multiple (N=559) vs. single jobholders (N=395) | 75.73 vs. 82.86 | 1.11 (0.88 – 1.40) | 1.11 (0.88 – 1.40) | 1.17 (0.93 – 1.48) |
| 271-365 | Multiple (N=470) vs. single jobholders (N=323) | 79.64 vs. 85.99 | 0.88 (0.65 – 1.19) | 0.90 (0.66 – 1.23) | 0.95 (0.69 – 1.29) |

CIP: cumulative incidence proportion, shows the percentages of individuals having returned to work within one year after injury CIP is calculated over full data and evaluated at indicated times; it is not calculated from aggregates shown at left.. HR: Hazard ratio; CI: Confidence interval; * Adjusted for MSD, gender, age, occupation, industry, previous claims, and firm size; ** Adjusted for variables in model 1, and weekly workdays preceding MSD eligible for compensation benefits
